# The Collaborative Cross as a Resource for Modeling Human Disease: CC011/Unc, a New Mouse Model for Spontaneous Colitis

**DOI:** 10.1007/s00335-013-9499-2

**Published:** 2014-02-01

**Authors:** Allison R. Rogala, Andrew P. Morgan, Alexis M. Christensen, Terry J. Gooch, Timothy A. Bell, Darla R. Miller, Virginia L. Godfrey, Fernando Pardo-Manuel de Villena

**Affiliations:** 1 Division of Laboratory Animal Medicine, University of North Carolina, Chapel Hill, NC USA; 2 Department of Genetics, University of North Carolina, Chapel Hill, NC USA; 3 Department of Genetics, Lineberger Comprehensive Cancer Center, and Carolina Center for Genome Sciences, University of North Carolina, Chapel Hill, NC USA

## Abstract

Inflammatory bowel disease (IBD) is an immune-mediated condition driven by improper responses to intestinal microflora in the context of environmental and genetic background. GWAS in humans have identified many loci associated with IBD, but animal models are valuable for dissecting the underlying molecular mechanisms, characterizing environmental and genetic contributions and developing treatments. Mouse models rely on interventions such as chemical treatment or introduction of an infectious agent to induce disease. Here, we describe a new model for IBD in which the disease develops spontaneously in 20-week-old mice in the absence of known murine pathogens. The model is part of the Collaborative Cross and came to our attention due to a high incidence of rectal prolapse in an incompletely inbred line. Necropsies revealed a profound proliferative colitis with variable degrees of ulceration and vasculitis, splenomegaly and enlarged mesenteric lymph nodes with no discernible anomalies of other organ systems. Phenotypic characterization of the CC011/Unc mice with homozygosity ranging from 94.1 to 99.8 % suggested that the trait was fixed and acted recessively in crosses to the colitis-resistant C57BL/6J inbred strain. Using a QTL approach, we identified four loci, *Ccc1*, *Ccc2,* *Ccc3* and *Ccc4* on chromosomes 12, 14, 1 and 8 that collectively explain 27.7 % of the phenotypic variation. Surprisingly, we also found that minute levels of residual heterozygosity in CC011/Unc have significant impact on the phenotype. This work demonstrates the utility of the CC as a source of models of human disease that arises through new combinations of alleles at susceptibility loci.

**Electronic supplementary material:**

The online version of this article (doi:10.1007/s00335-013-9499-2) contains supplementary material, which is available to authorized users.

## Introduction

Inflammatory bowel disease (IBD) is characterized as an improper immune response to the host’s intestinal microflora. Its etiology is believed to be multifactorial with contributions from both the environment and underlying host genetics (Xavier and Podolsky 2007). The number of genetic loci identified as being associated with this disease is increasing rapidly. A recent meta-analysis of human genome-wide association studies and immunochip data implicated as many as 163 different loci and potentially 300 candidate genes, demonstrating the genetic complexity underlying this disease (Jostins et al. 2012).

Current murine models capture some of the underlying pathways; however, given the genetic complexity of IBD, many more monogenic and polygenic genetic etiologies remain to be identified (Brandl and Beutler 2012). Current murine models of colitis can be induced by chemical treatment (dextran sodium sulfate or 2,4,6-trinitrobenzene sulfonic acid); introduction of an infectious agent (*Helicobacter hepaticus, Citrobacter rodentium, Trichuris muris*); genetic engineering; or a combination of these methods (Mizoguchi 2012; Levison et al. 2013). It is well established that genetic background has a significant impact on the degree of colitis induced by these interventions (Mähler et al. 1998, 1999; Esworthy et al. 2011a, b). Numerous studies have been conducted to identify genetic loci associated with this varied phenotype, particularly using one of the most popular colitis models, the interleukin-10 knockout mouse (Farmer et al. 2001; Borm et al. 2005; Bleich et al. 2010; Büchler et al. 2012). This approach has identified as many as 17 genetic loci contributing to the phenotype with the strongest and most consistent association attributed to a region termed the cytokine-deficiency-induced colitis susceptibility locus 1 (*Cdcs1*), residing on the distal segment of murine chromosome 3 (Buettner and Bleich 2013). Loci on chromosome 3 have also been shown as strain-dependent contributors to lesions in a *Trichuris muris*-induced model of colitis (Levison et al. 2013), a G-protein alpha inhibitory 2 chain (*Gnai2* −/−) mutant (Borm et al. 2005), T-bet(−/−).Rag2(−/−) double-deficient mice (Ermann et al. 2011), Gpx1(−/−).Gpx2(−/−) double-deficient mice (Esworthy et al. 2011a, b) and a *H. hepaticus*-induced colitis and colonic tumor model (Boulard et al. 2012). However, these studies focused on a relatively small number of common strains. Recent extensive characterization of the genomes of laboratory mouse strains has demonstrated the limitations of classical inbred strains for genetic analysis of polygenic traits and the value of wild-derived inbred strains (Keane et al. 2011; Yang et al. 2007, 2011). Furthermore, it would be useful to develop a mouse model of spontaneous colitis in the absence of chemical treatment, introduction of an infectious agent or directed mutagenesis.

The Collaborative Cross (CC) is a newly developed murine recombinant inbred (RI) panel created as a tool for genetic analysis of complex traits (Churchill et al. 2004; Threadgill et al. 2002). In contrast to standard recombinant inbred panels created by the brother-sister mating of F2 animals derived from two inbred strains, the CC adds significantly more genetic diversity by the inclusion of eight founder strains (Collaborative Cross Consortium 2012). The founder strains of the CC include five classical inbred strains (A/J, C57BL/6J, 129S1/SvImJ, NOD/ShiLtJ and NZO/HlLtJ) and three wild-derived strains that were selected to represent three *Mus musculus* subspecies (CAST/EiJ, PWK/PhJ and WSB/EiJ). The genomes of the CC founders have been deeply characterized and compared with those of other common mouse stocks (Keane et al. 2011; Yalcin and Flint 2012; Yang et al. 2011). CC lines were bred at three different locations from the eight founder lines (Chesler et al. 2008; Iraqi et al. 2008; Morahan et al. 2008). The US lines were started at the Oak Ridge National Laboratory in Tennessee and were relocated to the University of North Carolina in 2009 (Threadgill et al. 2011). The genome of incompletely inbred CC lines and distributable inbred CC strains has been characterized recently (Collaborative Cross Consortium 2012), and regular updates are available through the University of North Carolina Systems Genetics Core Facility (SGCF) web site (http://www.csbio.unc.edu/CCstatus/index.py).

Typically, a large set of RI lines from a given panel are used jointly for genetic mapping (Flint and Eskin 2012; Threadgill and Churchill 2012) and this has been a key focus of early uses of the CC (Aylor et al. 2011). Collectively, these studies have shown that the CC has broad variation across a wide range of phenotypes (Aylor et al. 2011; Bottomly et al. 2012; Ferris et al. 2013; Kelada et al. 2012; Phillippi et al. 2013). A complementary use of the CC is as a source of new mouse models of human diseases. Models may be identified as outliers in phenotypic screens or by the recognition of clinical signs of disease by the animal care staff and subsequent veterinary examinations or necropsies.

**Fig. 1:**
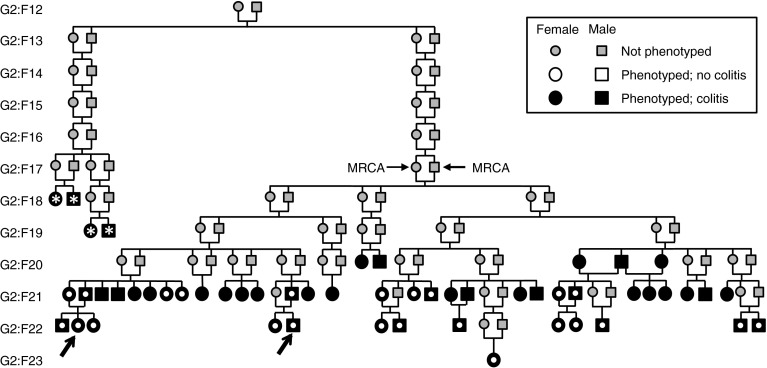
Pedigree of the CC011/Unc strain. Partial pedigree of the CC011/Unc strain including all phenotyped mice and their most recent common ancestors. Affected individuals with *asterisks* denote mice subject to full veterinary evaluation and pathogen analysis. Affected individuals with an* open circle* denote mice phenotyped using the “roll” method as described in the text. Most recent common ancestors (MRCA) of the CC011/Unc strain are indicated

**Fig. 2:**
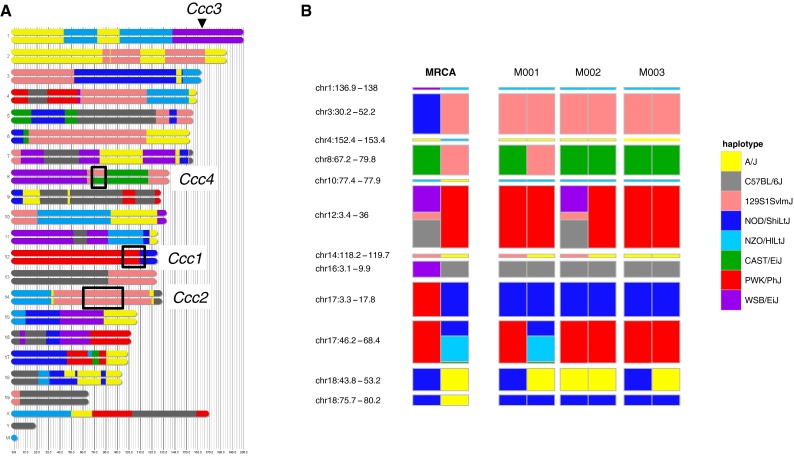
Founder haplotype contributions to CC011/Unc strain. **a** Genome mosaic of male M001.* Colors* denote the contribution of each founder strain according to the key to the* right* of the figure. *Black boxes* span the QTL identified in this study, *Ccc1*, *Ccc2* and *Ccc4*, while the* arrowhead* indicates the maximum peak at the third QTL, *Ccc3*. **b** Twelve intervals of residual heterozygosity in the CC011/Unc strain. Phased local haplotypes for the MRCA and the sires of the N2 population (M001, M002 and M003) are shown. The vertical widths of the intervals are to scale. Note M001, M002 and M003 have each fixed an allele for a different subset of the heterozygous regions

One such line, OR3252, was recognized as having an elevated incidence of rectal prolapses. Clinically, animals with mild prolapses maintained good body condition; however, eventually the prolapsed tissue became ulcerated or necrotic, or animals typically became hunched along with decreasing body condition scores and were euthanized. Necropsies of a sample of not only clinically affected but also unaffected animals revealed gross and histologic signs of diffuse, proliferative colitis. Remarkably, these lesions appeared in the absence of known murine pathogens. As the inbreeding threshold for distribution was reached, OR3252 was renamed CC011/Unc and deemed appropriate for genetic analysis (Figs. 1, 2). Here, we evaluate this new mouse inbred line as a potential model for IBD.

## Materials and Methods

### Animals and Housing

All animal work was performed according to the *Guide for the Care and Use of Laboratory Animals* under approved IACUC animal use protocols within the AAALAC accredited program at the University of North Carolina at Chapel Hill. OR3252 and CC011/Unc mice were obtained from the SGCF colony at UNC Chapel Hill, and C57BL/6J female mice used in the initial outcross were purchased from The Jackson Laboratory (Bar Harbor, ME). Dirty-bedding sentinels were tested quarterly and found to be negative for *Helicobacter* species by polymerase chain reaction (IDEXX RADIL) and seronegative for *Mycoplasma pulmonis*, CAR bacillus, ectromelia, epizootic diarrhea of infant mice (EDIM), lymphocytic choriomeningitis virus (LCMV), mouse adenoviruses 1 & 2, mouse hepatitis virus (MHV), mouse norovirus, mouse parvovirus, minute virus of mice, mouse polyoma virus, mouse cytomegalovirus, pneumonia virus of mice, Sendai virus, Theiler’s murine encephalomyelitis virus and reovirus 3 (IDEXX RADIL). Mice were group-housed in GM500 Green Line individually ventilated caging (Tecniplast, Buguggiate, Italy) with 70 air exchanges per hour. The room was maintained between 70 and 74°F with a 12-h light cycle. Cages were autoclaved as a unit containing Alpha-Dri^®^ bedding (Shepherd Specialty Papers, Watertown, TN), a cotton fiber Nestlet (Ancare, Bellmore, NY) and chow. The initial OR3252 mice received Prolab RMH 3500 or Purina LabDiet 5K20 breeder chow, but all animals described in the breeding experiment received autoclaved Purina LabDiet 5K20 breeder chow. Mice received autoclaved bottled RO water originating from a municipal source.

In addition to the dirty-bedding sentinel program, a subset of individual mice were selected for additional testing for *Helicobacter* genus, *Citrobacter rodentium* and mouse norovirus (Fig. 1). Feces were collected from live or dead animals to evaluate for *C. rodentium* via fecal culture. Fresh fecal material was plated onto MacConkey agar and incubated at 37 °C overnight and evaluated for colonies, and then if negative, it was incubated an additional 24 h and reevaluated. *Helicobacter* genus-level PCR was performed on frozen fecal samples by a commercial laboratory (IDEXX Radil). Serum samples were submitted for mouse norovirus testing via serology (IDEXX Radil).

The CC population is maintained and distributed by the SGCF at UNC at Chapel Hill (Welsh et al. 2012, http://csbio.unc.edu/CCstatus/index.py). Lines that reach 90 % homozygosity are ascertained by high-density genotyping (Collaborative Cross Consortium 2012; Yang et al. 2009) of the most recent common ancestors of the line (MRCA) and are made available to the public (Welsh et al. 2012). When lines reach this threshold, they are now called strains and are renamed according to the CC nomenclature. OR3252 was renamed CC011/Unc. Currently, the SGCF uses the second-generation Mouse Universal Genotyping Array (MegaMUGA) (Collaborative Cross Consortium 2012; Welsh et al. 2012) to determine the level of inbreeding and reconstruct the founder mosaic of every mouse and line. The details of the haplotype reconstruction will be published elsewhere (Chen Ping Fu and Leonard McMillan, personal communication).

The SGCF distributes strains in tiers. Tier 1 is a set of 12 strains that will stay consistent over time and (1) are at least 90 % homozygous, (2) represent all eight founders, (3) are in SPF or barrier status and (4) will be readily available from UNC. CC011/Unc is one of the twelve Tier 1 lines, and thus, its genome is a mosaic with contributions from each one of the eight founder strains. Over 94 % of the genome, all CC011/Unc mice are homozygous for the same founder allele (Fig. 2a). The regions that are segregating in the MRCA and the three males used to generate the backcrosses described in this report are shown in Fig. 2b. Genotypes and haplotype reconstruction for the CC011/Unc strain are available at http://www.csbio.unc.edu/CCstatus/index.py?run=AvailableLines.

To generate the main mapping population, a single CC011/Unc male mouse (named herein M001) was mated to six C57BL/6J females. Twenty-four F1 hybrid females were then backcrossed to the same M001 male to generate 257 N2 mice, to which we refer herein as the M1N2 population. Females were group-housed during breeding and parturition. Progeny were weaned into same-sex cages of up to five mice where they were housed until being necropsied at 20 weeks of age. Two additional backcrosses were generated in an analogous manner using two additional CC011/Unc mice (named herein M002 and M003) to generate the M2N2 (*n* = 85) and M3N2 (*n* = 66) populations, respectively. M001, M002 and M003 are siblings and littermates. All information regarding these mice is provided in Supplementary Table 1.

### Necropsy and Histologic Evaluation

Mice were euthanized with inhaled CO_2_ according to institutional policy and the AVMA Guidelines on Euthanasia prior to full gross necropsies being conducted. Whole colons were removed from the mouse, detached from the mesentery and opened via a longitudinal incision along the antimesenteric wall. Lumenal contents were carefully removed and stored at −80 °C for potential future analysis of microbial composition. Colons were then carefully rolled with the mucosal surface oriented internally (Whittem et al. 2010), secured with a 25-gauge needle and fixed in 10 % neutral-buffered formalin for 24-48 h before being bisected, routinely processed and paraffin-embedded, sectioned at 5 μm and stained with hematoxylin and eosin. Cut sections were evaluated and scored by a veterinarian experienced in mouse pathology. Samples were assigned a value ranging from 0 to 3 (0 = no lesions, 1 = mild, 2 = moderate, 3 = severe) based on severity in each of the following seven categories: glandular hyperplasia, goblet cell loss, leukocyte density in the mucosa and submucosa, extent of lesion, degree of vasculitis, number of crypt abscesses and mucinous lakes and extent of ulceration. A cumulative value of 21 denotes the maximal colitis score.

### DNA Preparation and Genotyping

Low molecular weight DNA was isolated from three CC011/Unc, eight C57BL/6J and 111 N2 mice by proteinase K digestion, followed by 2-propanol precipitation and 70 % ethanol washing. Approximately 2 mm of mouse tail was harvested, flash-frozen on dry ice and digested with proteinase K overnight at 65 °C. The following day, DNA was extracted using the Qiagen Puregene Gentra kit (kit no. 158389; Qiagen GmbH, Hilden Germany). Genotyping was performed with the MegaMUGA genotyping microarray (Neogen/Geneseek, Lincoln, NE), a new 78,000-probe array based on the Illumina^®^ Infinium platform to be described elsewhere.

### QTL Mapping

A list of 1059 maximally informative SNP markers from the MegaMUGA genotyping array was chosen for QTL mapping using the following criteria. First, markers were required to be segregating between CC011/Unc and C57BL/6J but not segregating within CC011/Unc or C57BL/6J. Second, only markers robustly called in >85 % of parental and M1N2 samples (*n* = 111) were selected. Finally, a subset of markers passing these quality controls was selected in order to create an evenly spaced grid across the genome with a median intermarker physical distance of 1.8 Mbp. Based on the known genotypes of the parental strains (http://www.csbio.unc.edu/CCstatus/index.py?run=AvailableLines), genotypes of M1N2 progeny were recoded as AA (homozygous for the CC011/Unc allele) or AB (heterozygous). Physical positions for each marker in the NCBIm37/mm9 genome assembly were converted to sex-specific genetic positions derived from the recombination map of the Heterogeneous Stock (Cox et al. 2009), using the Mouse Map Converter tool hosted by the Center for Genome Dynamics (http://cgd.jax.org/mousemapconverter/). Single-locus QTL and two-locus QTL scans were performed using Haley-Knott regression as implemented in R/qtl (Broman et al. 2003). Scans were repeated using the recombination map estimated from the current experiment with insignificant effect.

An additional set of 18 pairs of markers was selected which were each, as a pair, mutually informative between C57BL/6J and the two CC011/Unc haplotypes in regions where the M001 remains heterozygous (Supplementary Table 1). These markers were used for tests of association (one-way ANOVA) between the colitis phenotype and local haplotype in the segregating regions.

### Validation of QTL

In order to validate the QTL identified in this study, 146 additional M1N2 mice as well as all M2N2 (*n* = 85) and M3N2 (*n* = 66) mice were genotyped at three new markers tagging *Ccc1, Ccc2* and *Ccc3*. For *Ccc4*, samples were genotyped for at least one of the two pairs of markers that discriminate between the alleles segregating in the M001 male and C57BL/6J. We selected relatively large (>25 bp) insertions or deletions from the Sanger Mouse Genomes Project database (Keane et al. 2011, http://www.sanger.ac.uk/resources/mouse/genomes/) for which CC011/Unc is predicted to have a variant allele with respect to C57BL/6J. We then designed the following PCR primers to target these variants and scored samples by size separation on agarose gels:

- Ccc1_SAT, Forward: CATGAGGTCTGGTGCTGTG (100146314 to 100146332)
- Ccc1_SAT, Reverse: TTCTTATTTCTGAGACTGAGCCTC (100146501 to 100146524)
- Ccc1_DEL, Forward: GATGACTCACACTCGACCATAAG (100371160 to 100371182)
- Ccc1_DEL, Reverse: TTCTTGGAGCAGAGGAATGAC (100371522 to 100371542)
- Ccc2_SAT, Forward: AATTCCTTGGCTTCCCTGAG (64864485 to 64864504)
- Ccc2_SAT, Reverse: AATGATCCCAGGATGGTGG (64864716 to 64864735)
- Ccc3_DEL2, Forward: TCAGGCCCTGCACTCATA (162842542 to 162842559)
- Ccc3_DEL2, Reverse: CTTGGTGGTTCTGGCATTCA (162842906 to 162842925)
- Ccc4_70.8, Forward: GTGAAGAGCAAAGTGTACACAGC (70835845 to 70835867)
- Ccc4_70.8, Reverse: GATTATGACTGTGCTCTTGGCCCTTG (70836110 to 70836135)
- Ccc4_71.4, Forward: CTGAGTATTGACTGGCAGATCTAC (71460592 to 71460615)
- Ccc4_71.4, Reverse: GATTGCAAGACCTTTCTTAGCTGAGC (71460919 to 71460944)
- Ccc4_75.2, Forward: CTCTCCCTTGCCAGTCTTGGAC (75225887 to 75225908)
- Ccc4_75.2, Reverse: CAGCTCATCAGATTTGGCAGC (75226249 to 75226269)
- Ccc4_75.6, Forward: CCCAGAACTAGTTAGAAAAGCACAATC (75696002 to 75696028)
- Ccc4_75.6, Reverse: CATTATAAGCTAATATAGATCCCCCTTAAGGTTG (75696233 to 75696266)

PCR was performed on crude whole genomic DNA extracted by heating a 2-mm tail clip in 25 mM NaOH/ 0.2 mM EDTA for 60 min at 95 °C, followed by the addition of 40 mM Tris-HCl. The samples were then spun at 2,000 rpm for 10 min, and the supernatant collected for use as PCR template. PCR reactions contained 1.5-2 mM MgCl_2_, 0.2-0.25 mM dNTPs, 0.2-1.8 μM of each primer and 0.5-1 units of GoTaq polymerase (Promega) in a final volume of 10-50 μL. Cycling conditions were 94 °C, 4 min, 35 cycles at 94°, 55° and 68-72 °C for 30 s each, with a final extension at 68-72 °C, 7 min.

## Results

### Mice from the OR3252 Line and CC011/Unc Strain Show Pathologic Features of Colitis

Sporadic reports of rectal prolapses in the OR3252 line were observed as early as 2009 (generation G2:F7), shortly after the relocation of the CC to UNC (Threadgill et al. 2011). An increased incidence of prolapses prompted a full veterinary evaluation of four animals from generations G2:F18 and G2:F19 at ages 6 and 5 months, respectively (Fig. 1). All four animals were individually tested and found negative for intestinal parasites, *Helicobacter spp*. and *Citrobacter rodentium*. Due to concern that the *Citrobacter* organism may no longer be detectable late in the course of infection, feces from additional OR3252 colony animals at each week of age, from 4 through 16 weeks, were cultured and found to be negative.

**Fig. 3:**
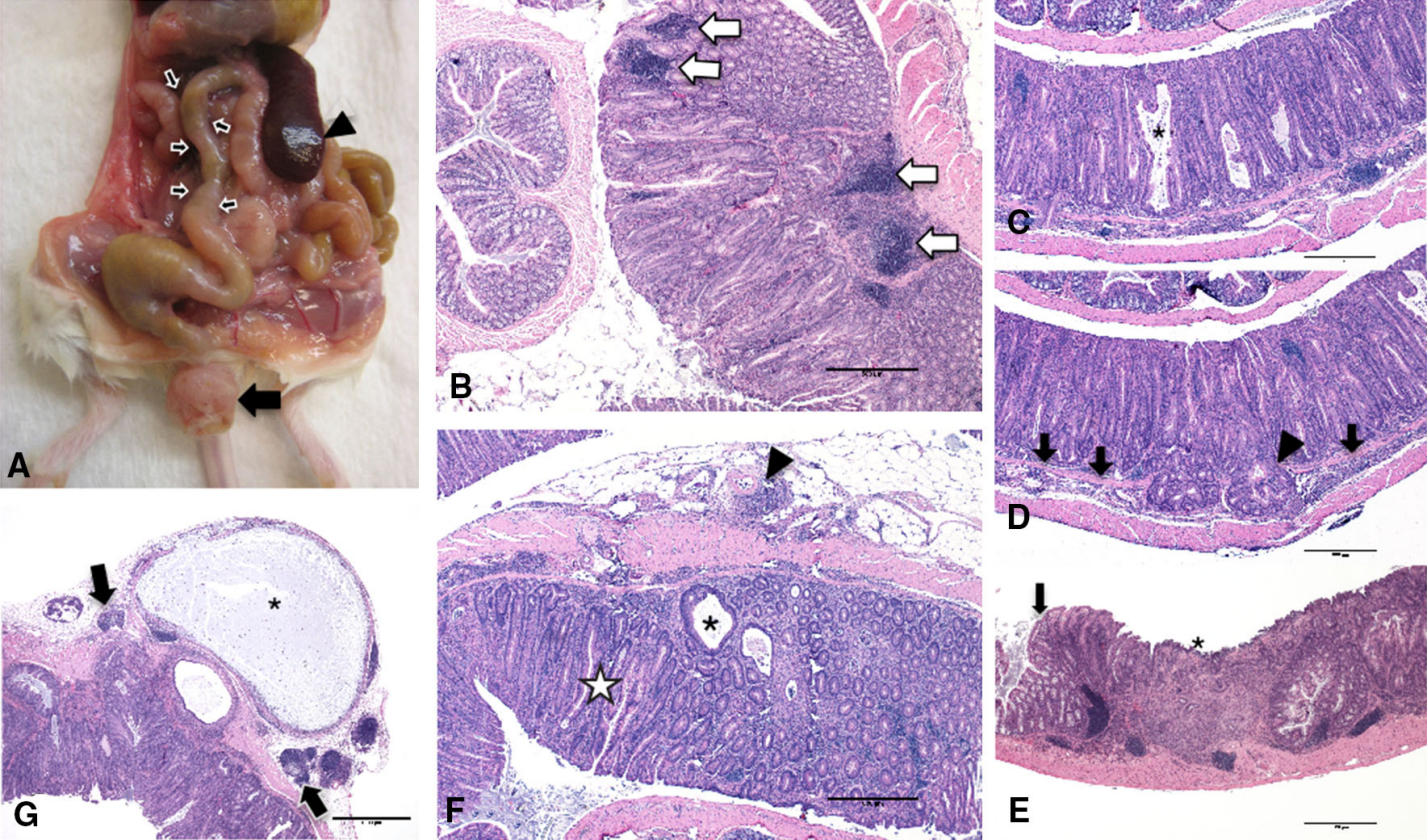
Gross and histologic lesions associated with colitis in CC011/Unc strain. **a** Gross necropsy of 20-week-old mouse. Note the enlarged spleen (*arrowhead*), thickened proximal colon (*small arrows*), relative lack of abdominal fat, and rectal prolapse (*large arrow*). **b** Focal colitis on transverse colonic fold (*right*) contrasted with unaffected transverse colonic fold in same histologic section (*left*). The abnormal mucosa exhibits marked hyperplasia with glands approximately 2.5–3× normal height, increased glandular tortuosity, loss of mucous-producing cells, and numerous lymphoid aggregates (*arrows*) and leukocytic infiltrate within both the lamina propria and the submucosa. **c** Proliferative colitis with numerous mucinous lakes (*asterisk*). **d** Proliferative colitis with submucosal herniation of the mucosal glands (*arrowhead*) through the muscularis mucosa (denoted by *arrows*). **e** Ulceration (*asterisk*) and focal colitis. The *arrow* denotes the region of demarcation between normal tissue (*left*) and the affected tissue. **f** Section from mouse with diffuse colitis depicting marked periarteritis (*arrowhead*), severe inflammation throughout the submucosa and lamina propria, marked glandular hypertrophy with significant reduction in the number of mucous cells (*star*), and mucinous lakes (*asterisk*). **g** Large mucinous serosal cyst (*asterisk*) with small reactive mesenteric lymph nodes on both sides (*arrows*)

Clinically, the animals were in adequate body condition with rectal prolapses with mild perineal fecal crusting and produced normally colored, soft, yet formed stool. Gross examination revealed a relative lack of abdominal fat, marked splenomegaly, enlarged mesenteric lymph nodes and a diffusely thickened colonic wall giving the colon a characteristic opaque “pipestem” appearance (Fig. 3a). No other gross abnormalities were noted.

Histologically, there was extensive mucosal hyperplasia characterized by loss of mucus cells, mucosal height often greater than three times that of normal colon, arborization of the glandular structures and infrequent glandular herniation into the submucosa (Fig. 3b–e). There were also few to many mucinous lakes and occasional crypt abscesses (Fig. 3c, f, g). The lamina propria was distended by a predominately lymphocytic mixed leukocytic infiltrate and increased numbers of lymphocytic aggregates (Fig. 3b–g). Inflammatory cells extended beyond the muscularis mucosa into the submucosa around serosal and mesenteric vessels, forming a necrotizing and suppurative or obliterative arteritis (Fig. 3f). The spleen and mesenteric lymph nodes showed marked lymphoid hyperplasia. The heart, thymus, lungs, liver, kidneys, adrenal glands and reproductive organs were unremarkable.

**Fig. 4:**
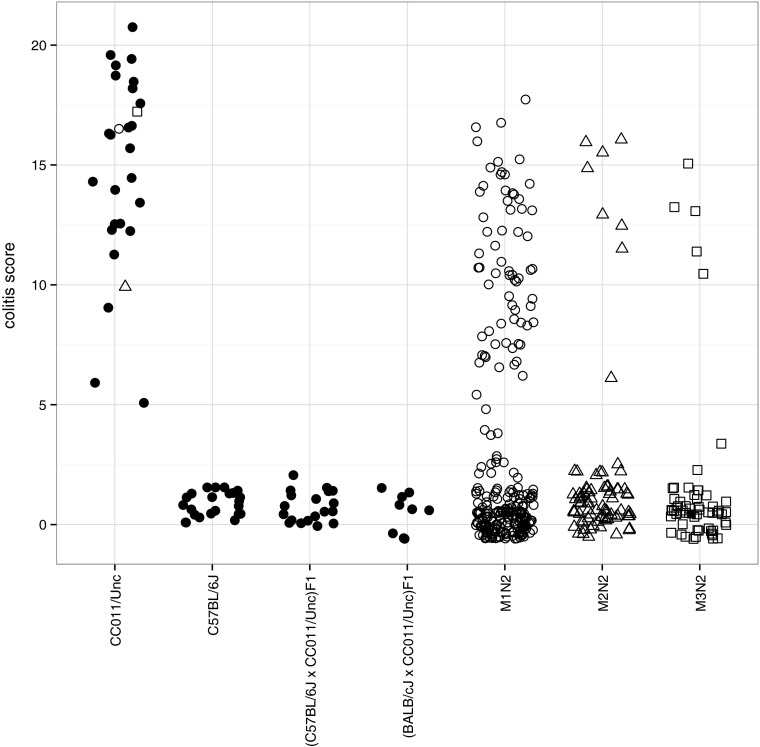
Colitis severity distribution in CC011/Unc, C57BL/6J, F1 and N2 progeny. Colitis score (*y*-axis) represents the sum of scores for seven components of the phenotype and ranges from 0 (no disease) to 21 (maximal disease). Each point represents one animal; *open circles*, animals which were scored for the phenotype and genotyped for genetic mapping, and *closed circles*, animals which were phenotyped but not genotyped. The CC011/Unc sires of the three groups of N2 progeny (M1N2, M2N2 and M3N2) mice are indicated with an *open circle*, *triangle* and *square*, respectively. Points are jittered in both* vertical* and* horizontal* directions for legibility

Prior to attempting to map loci associated with colitis in the CC011/Unc strain, we phenotyped a large cohort of mice from this strain aged 12-60 weeks, including mice from generations G2:F18 to G2:F23 and spanning the entire pedigree (Fig. 1). All mice phenotyped had gross and/or microscopic colitis and were assigned colitis scores. We also phenotyped 23 mice from the C57BL/6J inbred strain, 28 F1 hybrids derived from crosses between these two lines and 9 (BALB/cJ × CC011/Unc)F1 hybrids all of which were 20 weeks of age or older. As shown in Fig. 4, the colitis score is consistently higher in CC011/Unc mice than in C57BL/6J and F1 hybrids, while the scores of C57BL/6J and F1 hybrid mice are indistinguishable. We assumed that the colitis phenotype was fixed in CC011/Unc since the G2:F12 generation and that it behaved as a recessive trait in crosses to C57BL/6J and BALB/cJ.

### Genome Scans Identify Three Loci Associated with Colitis in the CC011/Unc Strain

**Fig. 5:**
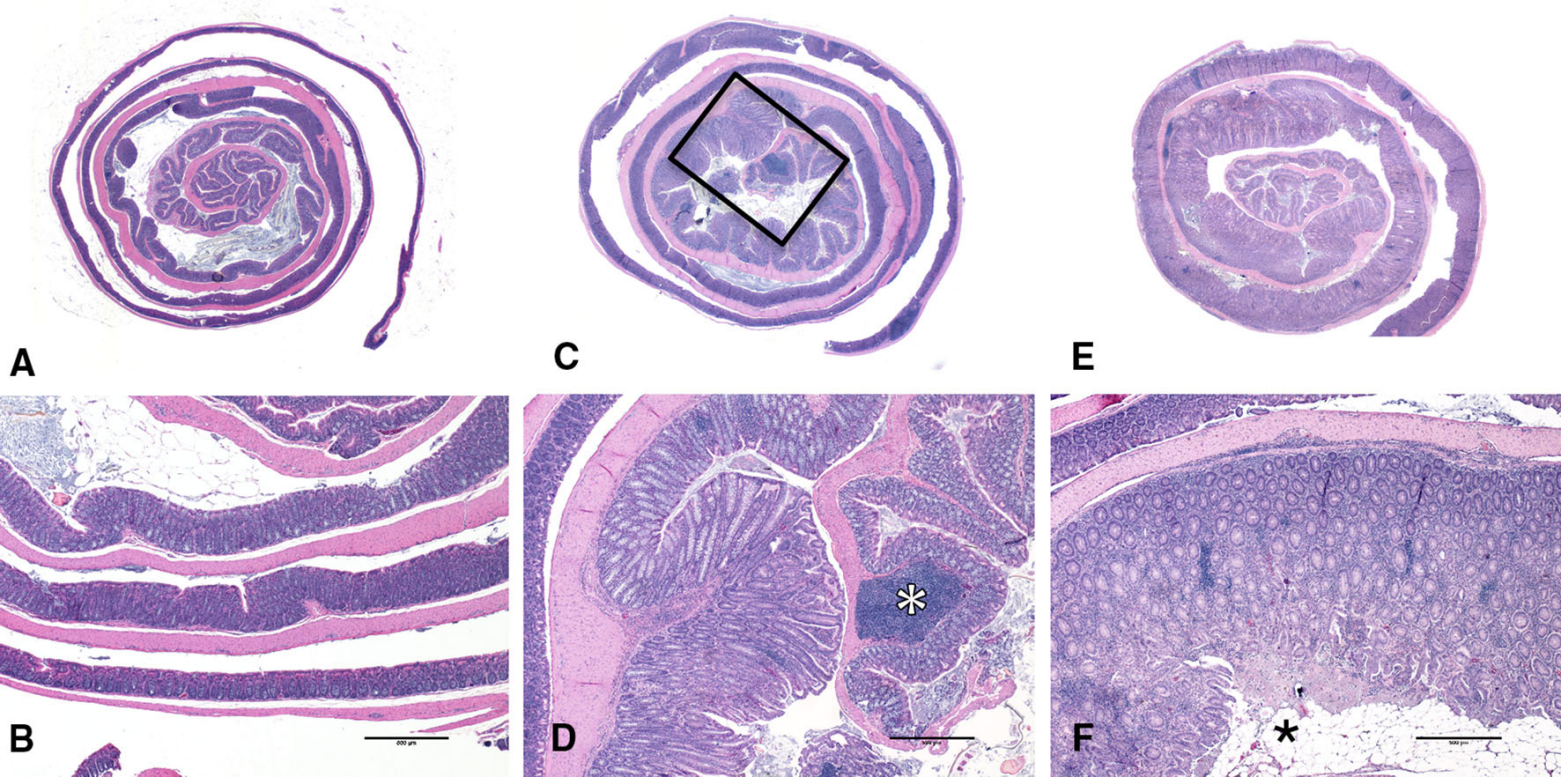
Range of histologic colitis in N2 mice. Photographs in the* top row* depict colon rolls at 1× magnification, while the* bottom row* depicts colons at a higher magnification. **a** An unaffected colon with a colitis score of zero paired with (**b**), a magnified region of the same colon. Note the uniformity and normal height of the mucosal glands as compared to the abnormal glands in (**d**) and (**f**). **c** A mildly affected colon (colitis score = 7) with a relatively focally affected region. The *black rectangle outlines* the region magnified in (**d**). Note the focal hyperplasia, decreased mucous cell population and inflammatory infiltrate within the lamina propria and submucosa. A large lymphoid aggregate appears on a proximal fold (*white asterisk*). **e**, **f** Represent severely affected colons (colitis score = 18). The entire length of the colon is diffusely thickened and inflamed (**e**). At higher magnification (**f**), severe hyperplasia, inflammatory infiltrate and superficial ulceration (*asterisk*) are evident

We generated, aged and phenotyped 257 N2 samples derived from a single CC011/Unc male, M001 (*i.e*., the M1N2 population). M001 is 98.85 % inbred but still segregating for four intervals throughout the genome (Fig. 2b). The distribution of clinical scores among the N2 is shown in Fig. 4. We selected 111 of these N2 samples (51 females and 60 males) for dense genotyping. Samples were selected among mice that were phenotyped for the colitis trait and represent a cross section of all clinical scores (Figs. 4, 5). Samples were genotyped using MegaMUGA array (see “Methods”). We selected 1059 SNP markers from the array that provided informative and reliable genotypes for QTL mapping in this backcross as described in “Methods.”

**Fig. 6:**
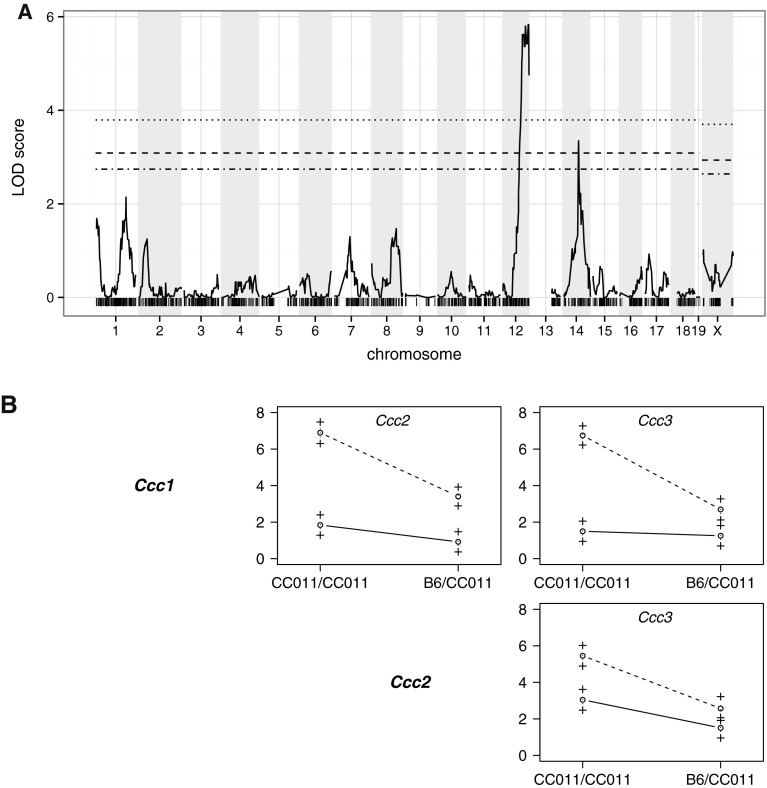
QTL scan for colitis score under single-locus model. **a** LOD score profile (*n* = 111 mice). Significance thresholds (*dashed-dotted line*, *α* = 0.10;* dashed line*, *α* = 0.05; *α* = 0.01) were derived from 1,000 permutations of the genotypes, performed separately for the autosomes and for the X chromosome.* Vertical hashes* along abscissa represent marker positions. **b** Allele-effect plots for pairs of colitis QTL (*n* = 257 mice). Phenotype means (+/− standard error) among individuals homozygous for the CC011/Unc allele (CC011/CC011) or heterozygous for the C57BL/6J and CC011/Unc alleles (B6/CC011) at the QTL* peak* indicated in the top of each cell are plotted according to their genotype (*dotted line* homozygous, *solid line* heterozygous) at the QTL* peak* indicated to the left of each row.* Parallel lines* are indicative of purely additive effects between loci, while convergent or *crossing lines* suggest epistasis

A single-locus genome scan identified two QTL that reached genome-wide significance at *α* = 0.05 (Fig. 6a). The most prominent QTL is located in distal chromosome 12, while the second QTL is located in the middle of chromosome 14; the loci were named *Ccc1* and *Ccc2*, respectively (for Collaborative Cross colitis locuses 1 and 2). For both QTL, homozygosity for the CC011/Unc allele is associated with higher colitis scores, while heterozygosity is associated with lower colitis scores (Table 2). A two-locus scan (Supplementary Figure 1) identified an additional locus on chromosome 1, named herein *Ccc3*. The pairwise allele-effect plots (Fig. 6b) indicate that *Ccc1* and *Ccc2* are additive. *Ccc3* and *Ccc2* appear to act epistatically with *Ccc1*. Globally, the additive effects of *Ccc1*, *Ccc2* and *Ccc3* and the *Ccc1* × *Ccc3* and *Ccc2* × *Ccc3* interactions explain 26.4 % of the observed variance in colitis score.

**Table 1:** QTL positions and local haplotypes

Locus	Chr	Peak (Mbp)	LOD interval (Mbp)	Haplotype
*Ccc1*	12	110.8	94.8-112.3	PWK/PhJ (proximal); NOD/ShiLtJ (distal)^a^
*Ccc2*	14	64.5	60.3-94.3	129S1SvlmJ
*Ccc3*	1	162.5	3.6-197.2	WSB/EiJ
*Ccc4*	8	70.5	67.2-79.8	CAST/EiJ; 129S1/SvImJ^b^

^a^CC011/Unc is recombinant with transition at 109.7 Mbp

^b^CC011/Unc is segregating for the indicated haplotypes in this interval

We then performed single-locus scans for each of the seven individual components of the colitis score (Supplementary Figure 2). The *Ccc1* locus was identified in each of these scans except for the crypt abscesses/mucoid cyst component. Interestingly, *Ccc3* reached significance for the ulceration component and was suggestive for the vasculitis component. A genome scan for normalized spleen weight also identified the *Ccc1* and *Ccc3* loci.

In contrast to crosses between standard inbred lines, the fact that the CC is a multiparental genetic reference population allows for an additional layer of genetic analysis, namely the identification of the local haplotypes associated with colitis severity at each one of the QTL. As shown in Fig. 2a, *Ccc2* is driven by the 129S1/SvImJ allele and *Ccc3* is driven by the WSB/EiJ allele. At the *Ccc1* locus, the CC011/Unc inbred line has a recombinant haplotype with a PWK/PhJ haplotype proximally and a NOD/ShiLtJ haplotype distally.

We also genotyped 147 additional N2 mice derived from the M001 sire at three QTL found in our genome scans. At each locus, there was a significant association of the CC011/Unc allele in homozygosity with higher colitis scores (Table 2). The differences in the magnitude of the effects can be explained by the fact that most of the highly affected individuals were genotyped with MegaMUGA and used in the initial genome scans. We also genotyped the progeny of the two additional backcrosses derived from male sibling littermates of male M001. *Ccc3* was replicated in both crosses, but to our surprise, neither *Ccc1* nor *Ccc2* could be confidently validated in the N2 populations derived from males M002 (population M2N2) and M003 (population M3N2) (Supplementary Table 2). *Ccc1* replicated but failed to reach statistical significance in the M2N2 population, while the allele effects at *Ccc2* were reversed in the additional N2 populations.

**Table 2:** QTL validation in the M1N2 population

Locus	Genotype	*n*	Score (mean ± SD)	*p*-value*
*Ccc1*	CC011/CC011	130	4.9 ± 5.6	2.65 × 10^−9^
	B6/CC011	127	1.4 ± 3.1	
*Ccc2*	CC011/CC011	119	4.2 ± 5.5	1.54 × 10^−3^
	B6/CC011	138	2.3 ± 4.2	
*Ccc3*	CC011/CC011	135	4.3 ± 5.7	9.66 × 10^−5^
	B6/CC011	122	2.0 ± 3.5	
*Ccc4*	CAST/129S1	69	4.4 ± 5.6	0.0212
	other	188	2.7 ± 4.5	

* Two-sample, two-tailed *t* test for difference in mean colitis score between genotype classes

### Regions Still Segregating in the CC011/Unc Strain have Significant Effects on Colitis

The lack of full replication of two major QTL found in our genome scan in backcrosses derived from very closely related male mice, combined with the phenotypic variation in both the initial backcross and the CC011/Unc strain as a whole (Fig. 4), prompted us to investigate the contribution of regions that are still segregating in CC011/Unc to the colitis phenotype.

**Fig. 7:**
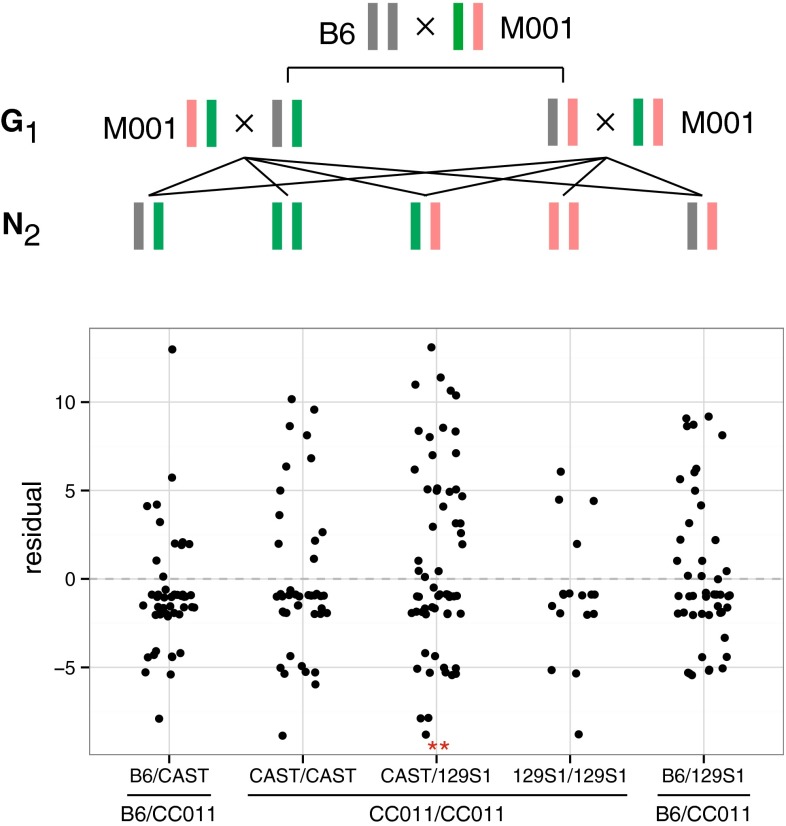
Relationship of residual heterozygosity in CC011/Unc to variability in colitis phenotype in N2 progeny. The sire (M001) of the 257 N2 progeny used for QTL mapping is segregating for the CAST/EiJ (CAST; *green*) and 129S1/SvImJ (129S1; *pink*) haplotypes at the *Ccc4* locus. The G1 progeny of a C57BL/6J (B6; *gray*) female and M001 forms two classes based on their diplotype at this locus: B6/CAST (*left*) and B6/129S1 (*right*). Four classes of N2 progeny can arise from each class of G1, of which three are shared. These classes are represented as color-coded chromosome segments in the* upper panel*. The outer two classes are treated as heterozygous (B6/CC011) for QTL mapping, while the inner three classes are nominally homozygous (CC011/CC011). The* lower panel* plots the residuals from the full three-QTL model (additive effects of *Ccc1*, *Ccc2* and *Ccc3* plus the *Ccc1* **×** *Ccc3* interaction) according to diplotype at *Ccc4* for all *n* = 257 M1N2 mice genotyped. Addition of diplotype at *Ccc4* to the regression model is significant only for the CAST/129S1 class (*t* = 2.185 on 1 df; *p* = 0.0298**). Presence of the CAST/129S1 diplotype at this locus adds 2.3 (95 % confidence interval 0.2, 4.4) points to colitis score

We tested whether inheritance of either one of the two haplotypes segregating at each of the four regions of residual heterozygosity in the M001 male (on chromosomes 8, 14, 17 and 18, see Fig. 2) is associated with the phenotype. This analysis identified a fourth locus on chromosome 8, *Ccc4*, associated with colitis in CC011/Unc (Table 1). At *Ccc4*, M001 is heterozygous for CAST/EiJ (CAST; green in Fig. 7) and 129S1/ImSVJ (129S1; pink in Fig. 7) haplotypes over a 16.5 Mbp region of chromosome 8. As shown in Fig. 7, the G1 progeny of a C57BL/6J (B6; gray) female and M001 form two classes based on their diplotype at this locus: B6/CAST (left in Fig. 7) and B6/129S1 (right in Fig. 7). Four classes of N2 progeny can arise from each class of G1, of which three are shared: B6/CAST, B6/129S1 and CAST/129S1. Of the five total progeny classes, three are treated as nominally “homozygous” (CC011/CC011) in a standard backcross analysis, but in fact, heterozygosity for the CAST/EiJ and 129S1/SvImJ alleles at *Ccc4* is associated with higher colitis scores, while neither allele has detectable effect in homozygosity (Fig. 7). We conclude that the relatively small fraction of the genome that remains heterozygous after 23 generations of sibling-mating may still exert profound influence on the phenotype. Note also that M002 and M003 are homozygous for the CAST/EiJ allele at *Ccc4* (Fig. 2b), a finding that may explain the failure to replicate some of our QTL in their N2 progeny.

During the phenotyping of the N2 mice, we also recorded body weight, liver weight and colon length (Supplementary Figure 2). A genome scan identified a locus on chromosome 17 associated with body weight. This locus, named *Ccbw1 (*for collaborative cross body weight locus 1), is associated with the NOD/ShiLtJ haplotype. No other QTL were identified in these scans.

## Discussion

CC011/Unc mice exhibit a chronic proliferative colitis with pathologic features similar to human IBD (Kumar et al. 2005). This colitis is characterized by a profound epithelial hyperplastic response with intralesional loss of goblet cells, increased tortuosity of glandular structure, multifocal ulceration and mucosal lymphoid hyperplasia (Fig. 3). The biology of the CC011/Unc strain emulates the human disease in that mice from this strain have a genetic predisposition to colonic inflammation in the absence of a chemical or immunological manipulation or infection with a known pathogen.

The initial clinical presentation and gross thickening of the colonic wall prompted suspicion of an intestinal pathogen in this line, most notably *Citrobacter rodentium* or a *Helicobacter* species. Both organisms cause a proliferative colitis histologically very similar to that seen in the CC011/Unc strain but with a different anatomic distribution. The lesions in the CC011/Unc strain tend to originate in the transverse colon just distal to the final transverse fold and then extend both proximally and distally as the lesions progress (Fig. 5). In contrast, lesions associated with *Helicobacter* tend to occur in the cecum and proximal colon, whereas *C. rodentium*-associated lesions occur in the distal colon. Extensive testing of both colony animals and dirty-bedding sentinels has ruled out these pathogens. Additionally, we ruled out intestinal helminth and protozoal parasites in the initial necropsy subjects, and although the agent is not known to cause disease in immunocompetent mice, animals are consistently monitored for mouse norovirus and have remained negative. We cannot exclude the possibility that the CC011/Unc strain harbors an unidentified intestinal pathogen. However, the C57BL/6J and BALB/cJ mice mated with the CC011/Unc male to produce the F1 generation showed no gross or histologic signs of colitis, and multiple CC lines co-housed with CC011/Unc mice long term in other experiments have shown no clinical signs of colitis (results not shown).

Even if a pathogenic organism is identified in this line in the future, the profound phenotype makes it a valuable model for studying the mechanisms underlying colonic inflammation. It is well established that immune tolerance in the gut is the product of complex interactions between microorganisms and both the innate and adaptive arms of the host immune system (Mueller and Macpherson 2006) and that IBD represents a departure from this balance. Although microbiological testing argues against a role for specific pathogens in the etiology of the CC011/Unc colitis phenotype, some portion of the residual variance is likely due to individual variation in the commensal gut flora. Co-housing (Campbell et al. 2012) and the maternal microflora (Palmer et al. 2007) are key determinants of this variation. Future studies should explore the relationship between environmental and microbiological covariates and severity of colitis in CC011/Unc.

One of the promises of the CC was its role as a source of new models of human disease. This expectation was predicated on the idea that new combinations of alleles at multiple loci in a multiparental recombinant inbred strain panel derived from eight highly diverse founder strains would produce some phenotypic outliers. The work reported here represents the definitive proof of principle for this concept. As expected, colitis in CC011/Unc depends on a combination of alleles at a minimum of four loci that is not found in any existing laboratory stock genotyped at high density with the Mouse Diversity array (Yang et al. 2011; Wang et al. 2012). The susceptibility loci are enriched for wild-derived alleles (PWK/EiJ at *Ccc1*; WSB/EiJ at *Ccc3*; and CAST/EiJ at *Ccc4*) as expected, given the high levels of the genetic diversity that they bring to mouse resources (Didion and Pardo-Manuel de Villena 2013). The loci we identified on chromosomes 12 and 14 (*Ccc1* and *Ccc2,* respectively) have not yet been described in studies investigating QTL associated with a strain-dependent predisposition to colitis. The large *Ccc3* locus potentially overlaps previously described colitogenic intervals identified on chromosome 1 including the IL-10 deficiency model (*Cdcs2*, peak marker 33.31 cM), G-protein-deficiency-induced colitis (*Gpdc2*, peak marker 50.78 cM) and the *Trichuris muris*-induced colitis model (*Tm1*, peak marker between 68.58 and 76.73 cM) (Farmer et al. 2001; Borm et al. 2005; Levison et al. 2013). At both the *Cdcs2* and *Gpdc2* loci, the underlying background strain is C3H/HeN, whereas the AKR/OlaHsd strain contributes to the allele at the *Tm1* locus. In the CC011/Unc strain, *Ccc3* encompasses regions of both classical (A/J and NZO/HILtJ) and wild-derived (WSB/EiJ) origin. The large size and the complex nature of identity by descent in classical inbred strains (Yang et al. 2011; Wang et al. 2012) preclude a meaningful discussion on potential candidate genes shared by the different models. Analysis of the IL-10 deficiency model of colitis also identified a contribution of the C57BL/6J haplotype on chromosome 8 (*Cdcs4*, peak marker *D8Mit191* at 21 cM) which main effect was limited to typhlitis and not colitis (Farmer et al. 2001). The location, mode of action and allele effects make it unlikely that *Cdsc4* and *Ccc4* loci are shared. We conclude that the CC and its complementary resource the Diversity Outbred population (Churchill et al. 2012; Svenson et al. 2012) are outstanding resources for modeling and genetically dissecting traits related to human health.

The diversity of modes of action of alleles at these loci is also noteworthy. *Ccc1* and *Ccc2* appear to act additively, while *Ccc3* has epistatic interactions with both *Ccc1* and *Ccc2*. Finally, alleles at *Ccc4* either are overdominant or have parent-of-origin effects. Our relatively small sample size precludes discrimination between these two modes of action, and the lack of reported imprinted loci on mouse chromosome 8 does not provide additional support for the second model. Despite these limitations, our results provide additional evidence for the remarkable complexity of the genetic architecture of biological traits in experimental crosses involving the laboratory mouse (Shimomura et al. 2001). Importantly, our results also indicate that human GWAS—which can identify only loci with additive effects—are unlikely to uncover the full spectrum of genetic determinants of human diseases.

Like most complex traits, colitis has genetic and non-genetic components. Using a pedigree-based variance-components approach, we estimate that the combination of additive genetic effects and cage effects accounts for 85.2 % of the variance in colitis score in the CC011/Unc strain. The fact that the phenotypic variance is approximately equal among affected individuals in the CC011/Unc strain and among N2 progeny homozygous for the CC011/Unc allele at *Ccc1*, *Ccc2* and *Ccc3* at first led us to assume that non-genetic factors were in part responsible for the variance in severity of the colitis phenotype in the CC011/Unc strain and in the backcrosses. It would be easy to conclude that although these three loci nominally capture 26 % of total phenotypic variance, this in fact represents a majority of the genetic component of the variance. Interestingly, colitis score has higher mean and greater variance in males than in females, even after accounting for genotype at *Ccc1*-*Ccc3*. Recasting the phenotype score as a (Poisson-distributed) count of non-zero components, females are substantially more likely to have a score of zero (odds ratio 2.3; 95 % confidence interval 1.3-4.0) and the effects of *Ccc1*–*Ccc3* are still significant in zero-inflated Poisson regression.

The lack of consistent replication of the QTL findings in two related backcrosses motivated a deeper analysis of this issue (Fig. 4; Supplementary Table 2). Remarkably, one of the four regions segregating in male M001 but not in males M002 and M003 harbors a locus, *Ccc4*, with an overdominant or parent-of-origin-dependent effect on the phenotype, which explains an additional 1.6 % of phenotypic variation. Users of the CC (and of many other resources such as congenics) will be well served by genotyping at high density the mice used as progenitors in their experiments. Such information can be used to frame their conclusions and to serve as a starting point for additional studies. For example, the CC011/Unc strain is currently maintained in four colonies that may have fixed different alleles at regions segregating in the pedigree (Figs. 1, 2). Three of these colonies, including those at The Jackson Laboratory, MMRRC-UNC and the Division of Laboratory Animal Medicine at UNC, have undergone severe bottlenecks which have in fact created substrains that differ at a small percentage of their genome (for example, the CC011/UncJ subline is fixed for CAST/EiJ alleles at *Ccc4*). These substrains may have effectively mendelized some of the loci with small and complex effects on colitis, opening an avenue for their identification in appropriately designed studies. Geneticists are well aware that substrains that differ phenotypically but are almost identical genetically can be powerful tools for the genetic analysis of complex traits (Kumar et al. 2013). All substrains of CC011/Unc have the colitis phenotype and are unlikely to have diverged much in regions that were fixed in the MRCA (Figs. 1, 2). Thus, any trait difference that is genetic can be ascribed to the different combinations of alleles at segregating regions. In other words, a perceived weakness of the incomplete inbred nature of the current CC lines can be turned into an advantage for the genetic analysis of complex traits by screening for consistent phenotypic differences within CC lines that can not easily be accounted by environmental variation.

We conclude that the CC011/Unc strain represents a valuable model of spontaneous colitis through both its phenotype and combination of newly described colitogenic loci. Additionally, we have successfully shown the value of the CC as a source of new models of human disease.

## Electronic supplementary material

Below is the link to the electronic supplementary material.

QTL scan for colitis score under two-locus model. Color in a cell (x,y) above the diagonal represent LOD score under a full model considering both additive and epistatic effects between the loci x and y. Color in a cell (y,x) below the diagonal represents LOD score for epistatic effects between the loci y and x, conditional on the additive effects. The color scale for the upper triangle of the matrix is indicated at the left of the scale bar, and the scale or the lower triangle to the right. (PDF 5,434 kb)

QTL scans for all phenotypes under single-locus model. Significance thresholds (dashed-dotted line, *α* = 0.10; dashed line, *α* = 0.05; *α* = 0.01) were derived from 1,000 permutations, performed separately for the autosomes and for the X chromosome. Vertical hashes along abscissa represent marker positions. (PDF 262 kb)

Residual phenotypic variation within genotype classes at *Ccc1*, *Ccc2* and *Ccc3*. Colitis score (*y-*axis) within two-locus genotype defined by *Ccc1* and *Ccc2*, coded as AA (homozygous; CC011/CC011) and AB (heterozygous; B6/CC011). Filled circles denote individuals homozygous at the epistatic locus *Ccc3* and open circles denote individuals heterozygous at *Ccc3*. (PDF 33 kb)

Raw phenotype and genotype data. Missing data are coded as “NA.” Column legend is provided as an additional spreadsheet tab. (XLSX 1,808 kb)

Test for association between colitis severity and genotype at the *Ccc1*, *Ccc2* and *Ccc3* loci in the backcrosses derived from males M002 and M003, respectively. (XLSX 10 kb)
