# Supplementary figures and images for: The Collaborative Cross as a Resource for Modeling Human Disease: CC011/Unc, a New Mouse Model for Spontaneous Colitis
Source: Mamm Genome. 2014 Feb 1;25(3):95–108. doi: 10.1007/s00335-013-9499-2 (PMC3960486; doi:10.1007/s00335-013-9499-2)

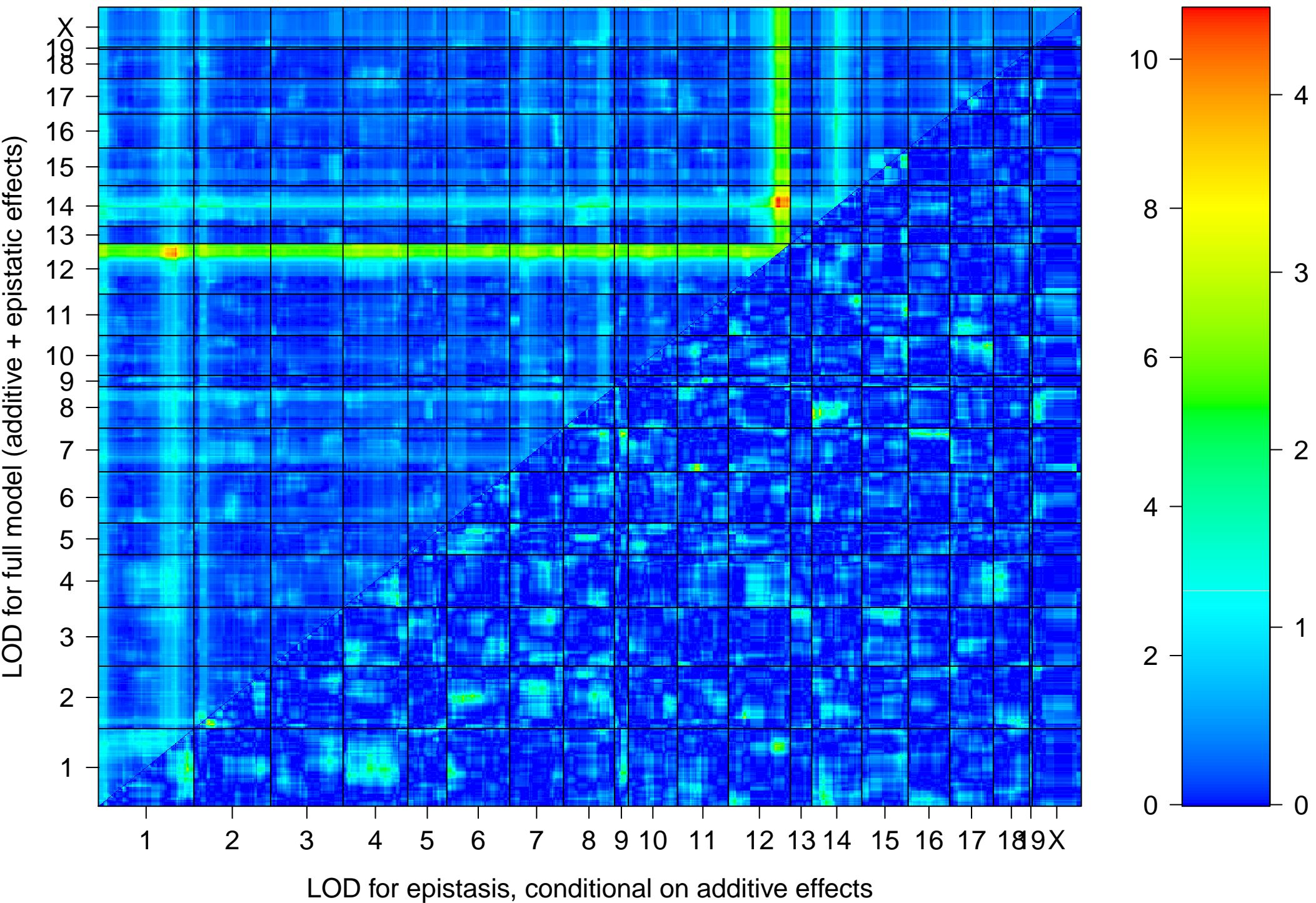

Supplement: Supplementary file 1 — QTL scan for colitis score under two-locus model. Color in a cell (x,y) above the diagonal represent LOD score under a full model considering both additive and epistatic effects between the loci x and y. Color in a cell (y,x) below the diagonal represents LOD score for epistatic effects between the loci y and x, conditional on the additive effects. The color scale for the upper triangle of the matrix is indicated at the left of the scale bar, and the scale or the lower triangle to the right. (PDF 5,434 kb) [file 335_2013_9499_MOESM1_ESM.pdf]

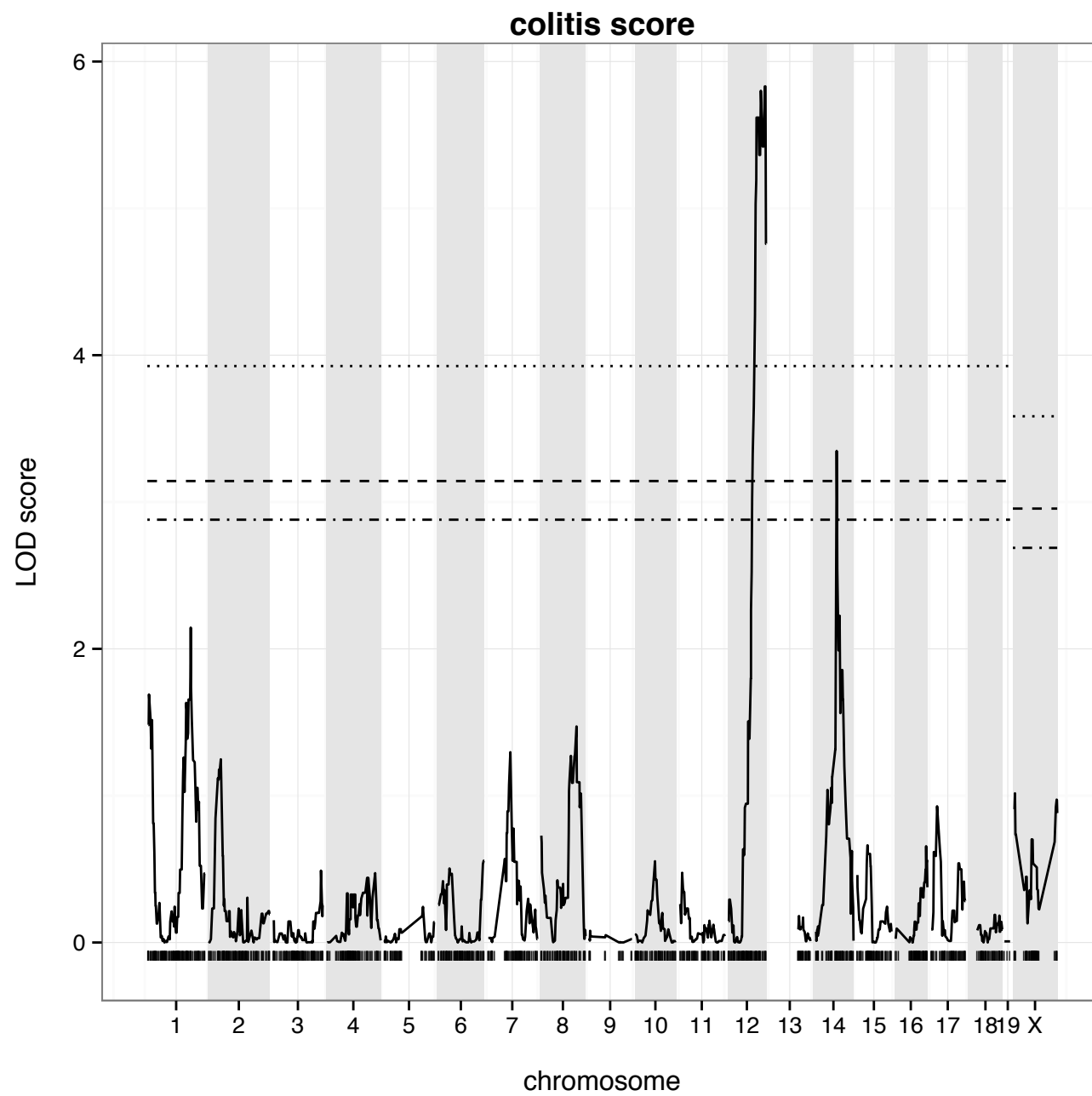

# hyperplasia subscore

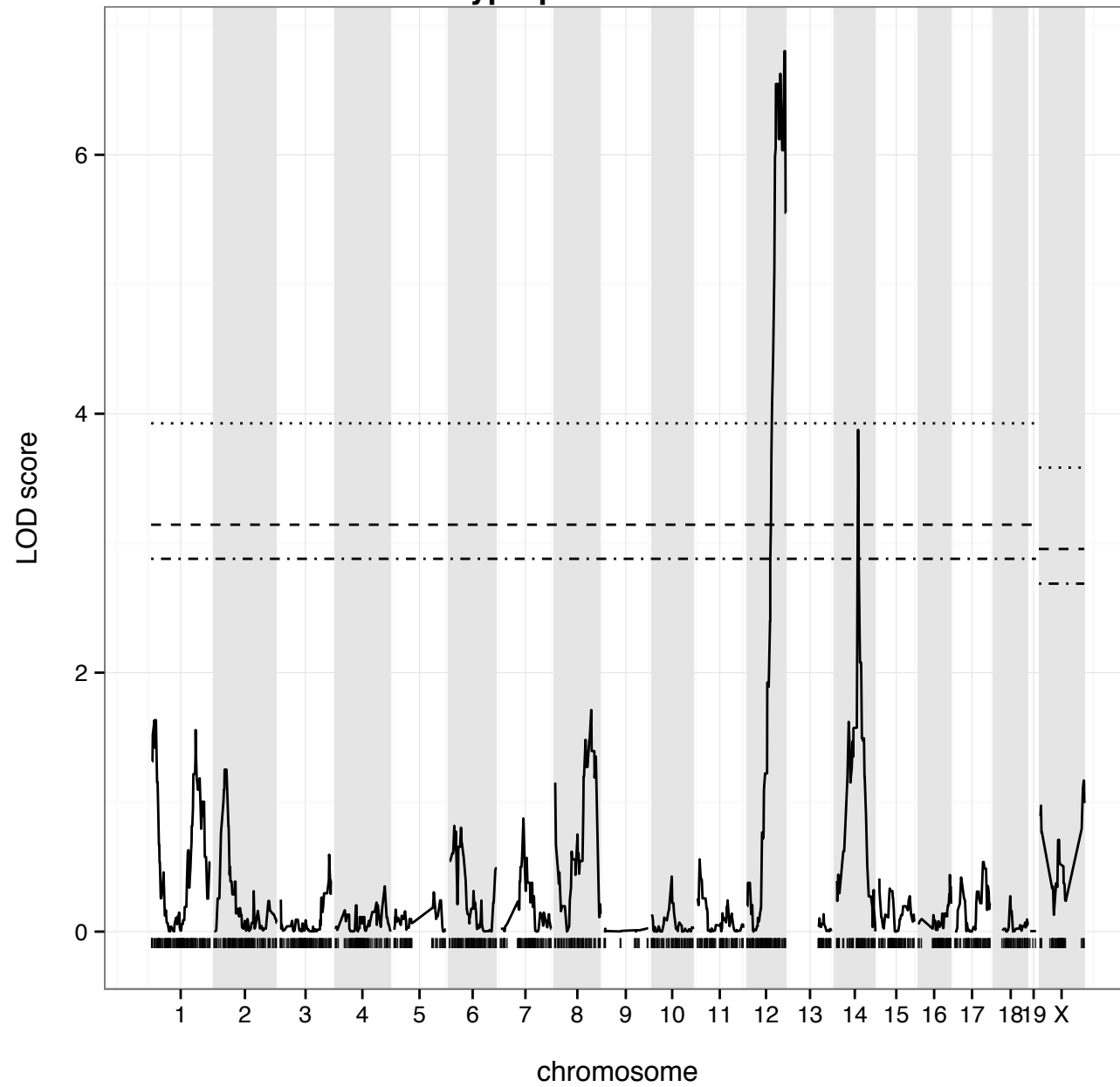

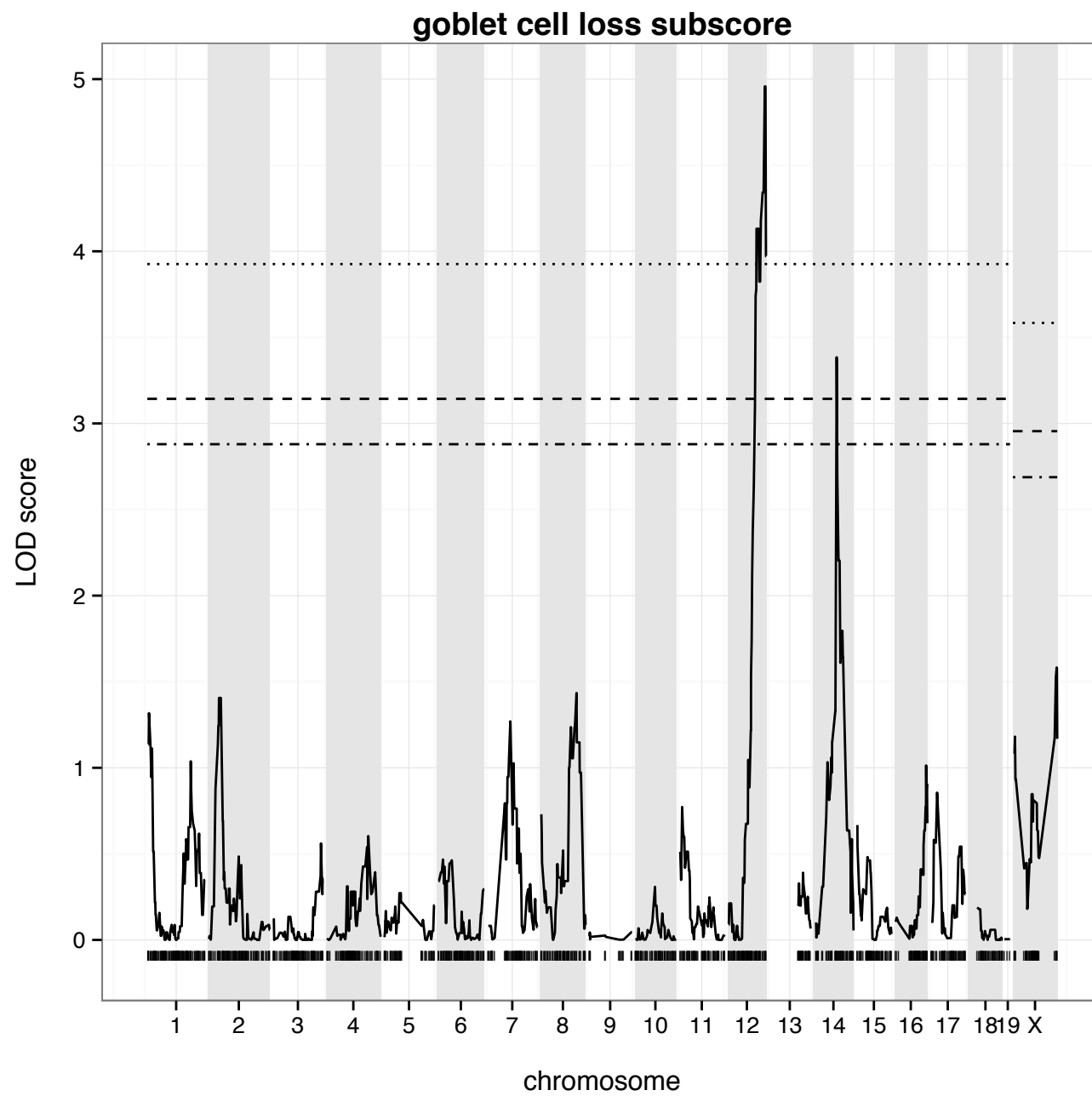

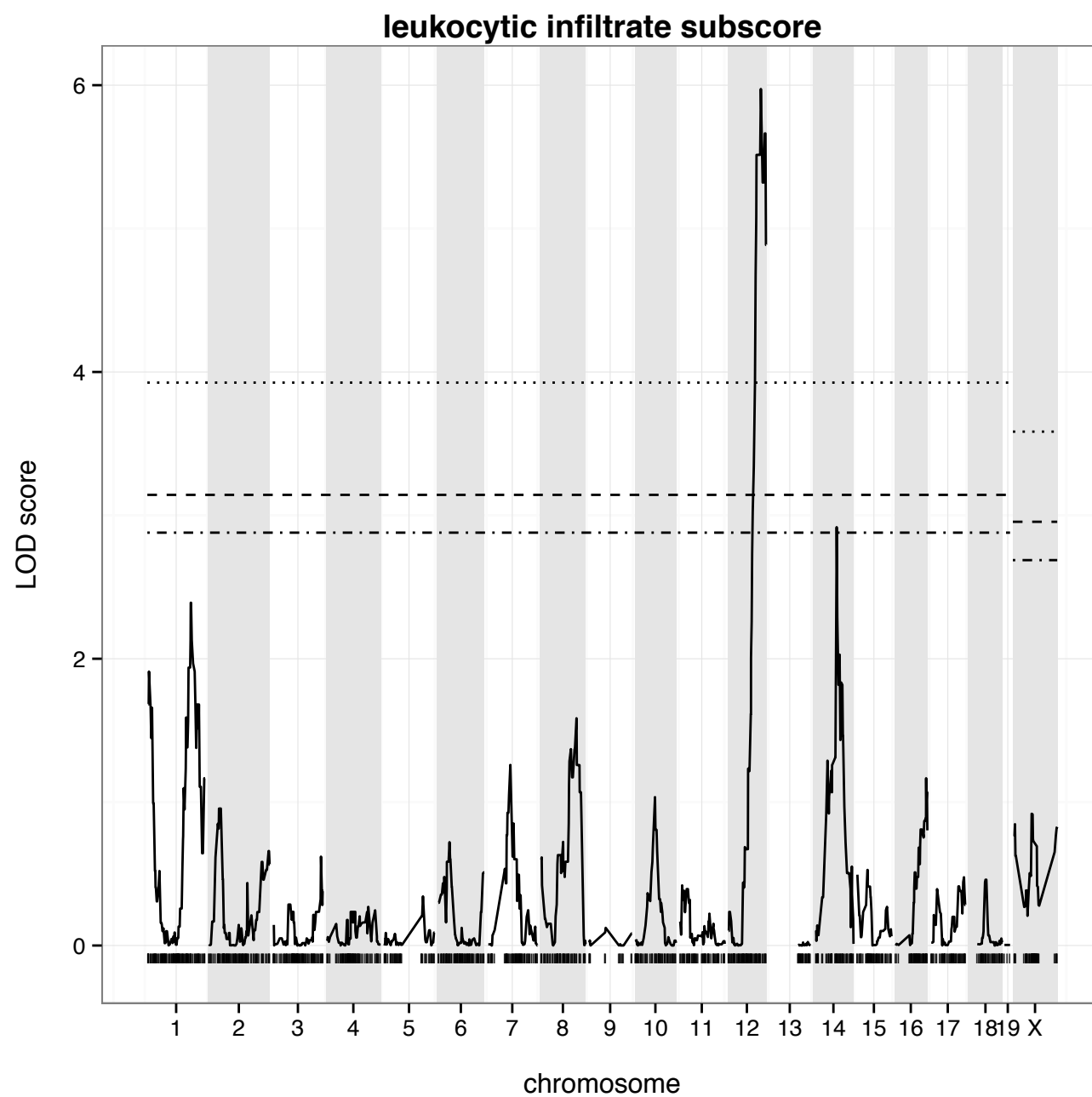

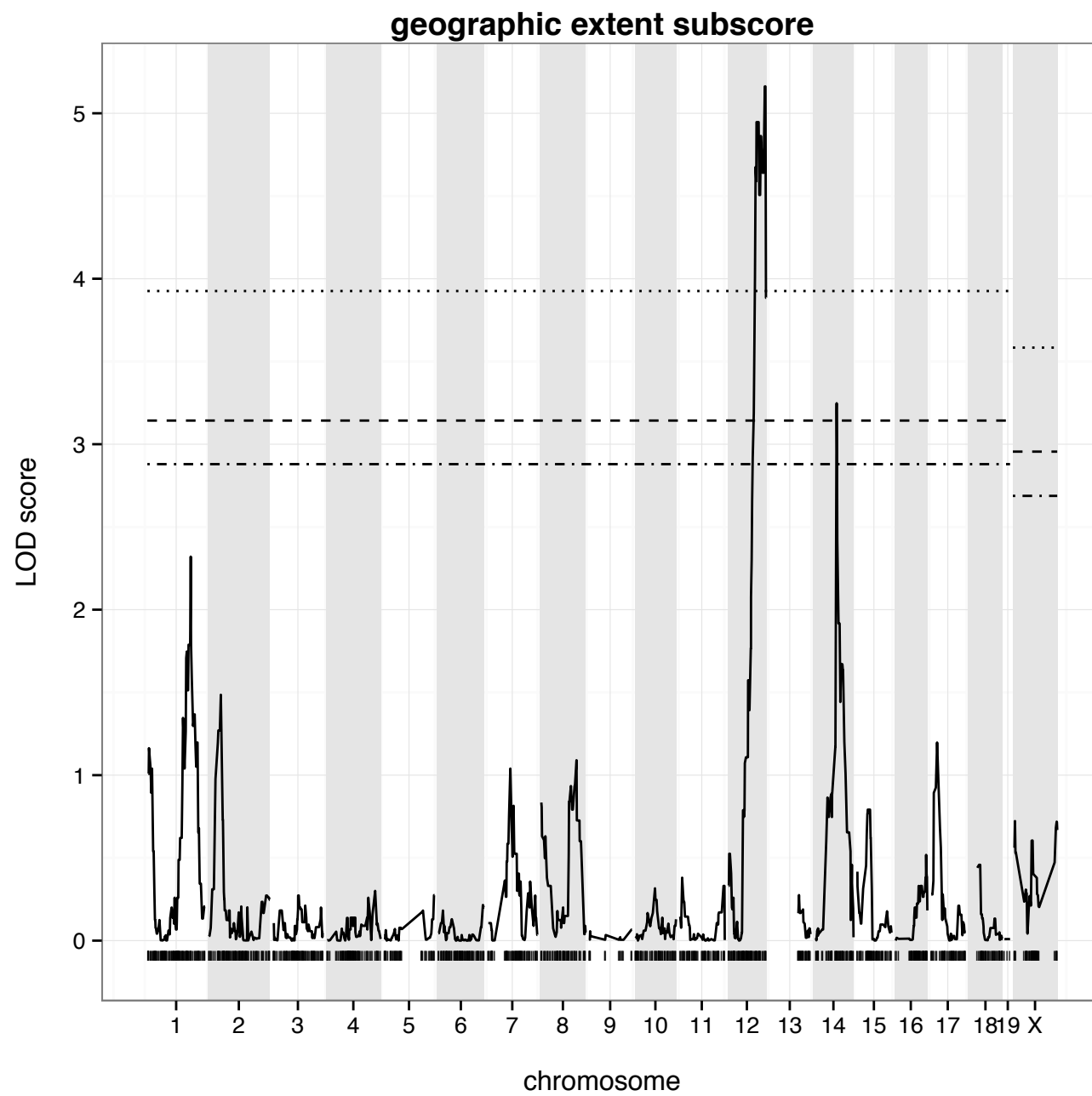

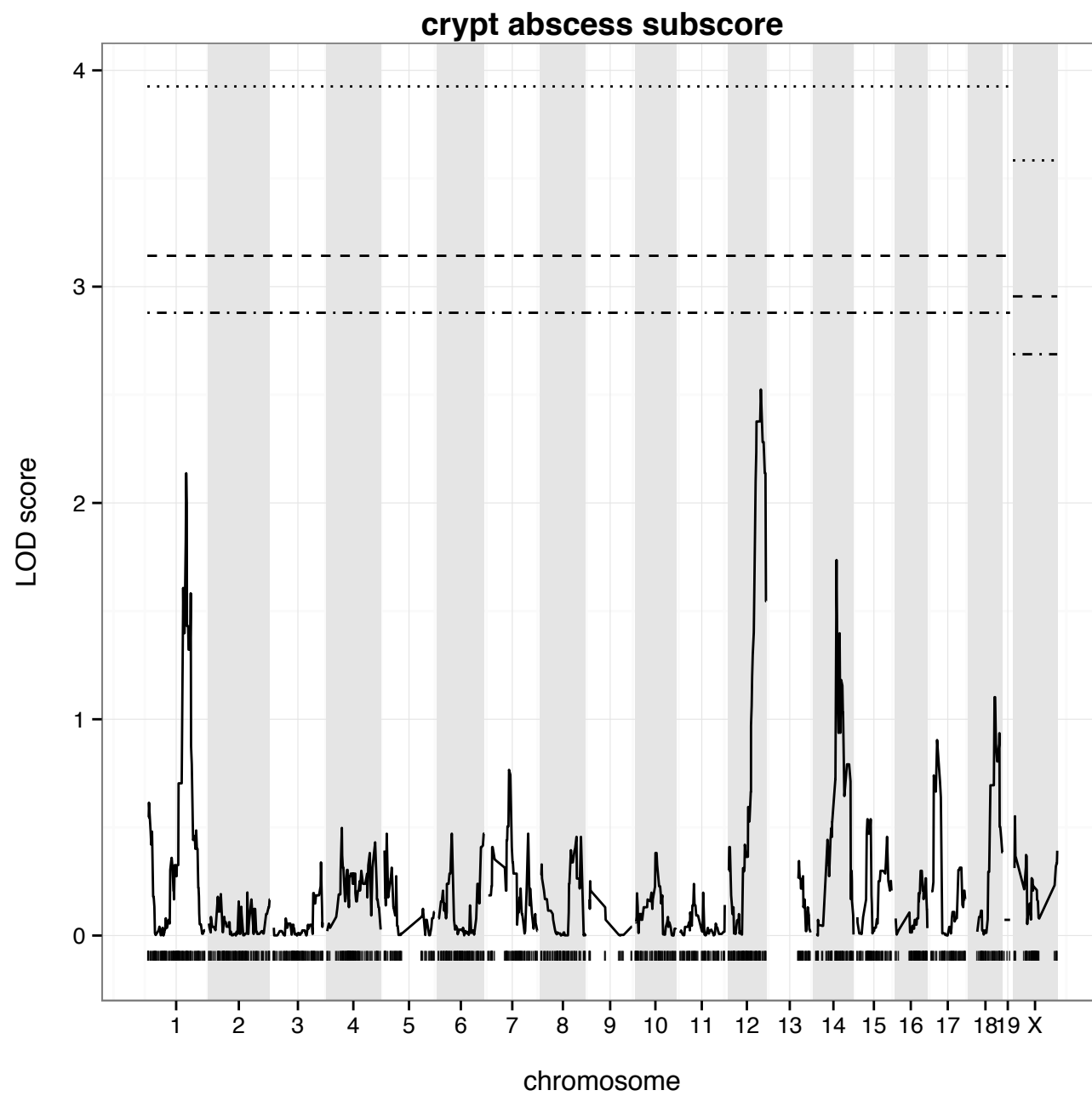

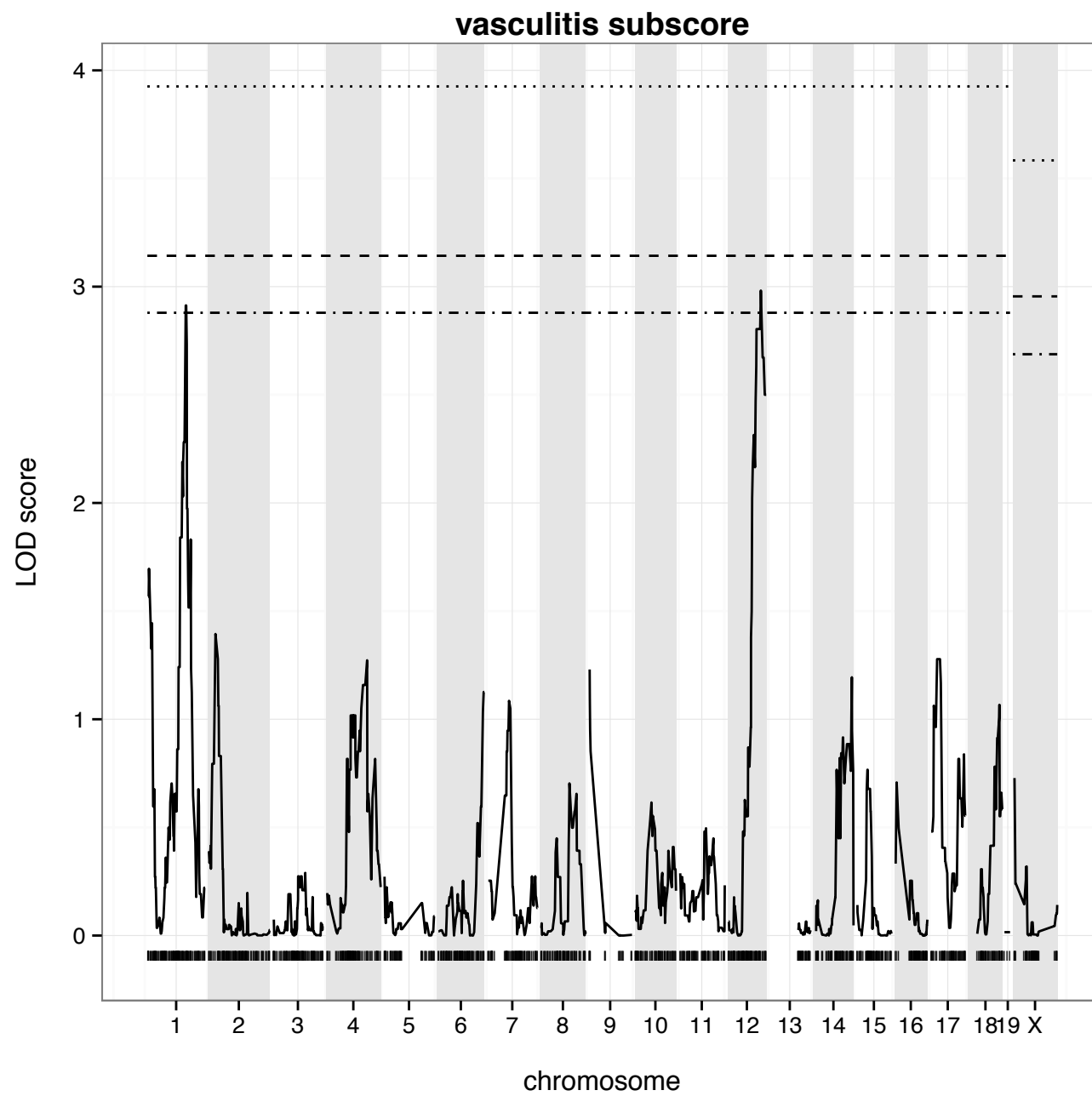

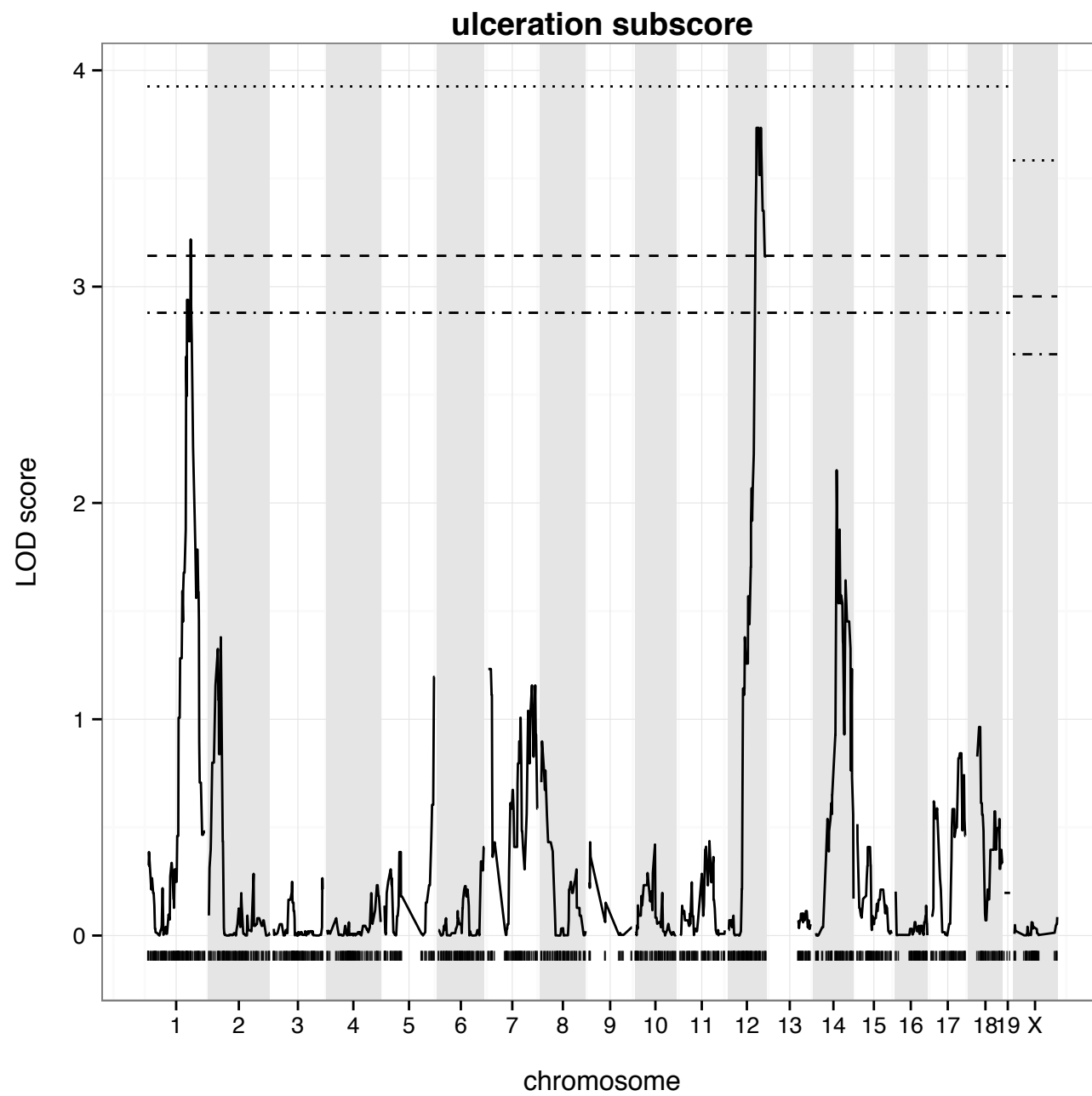

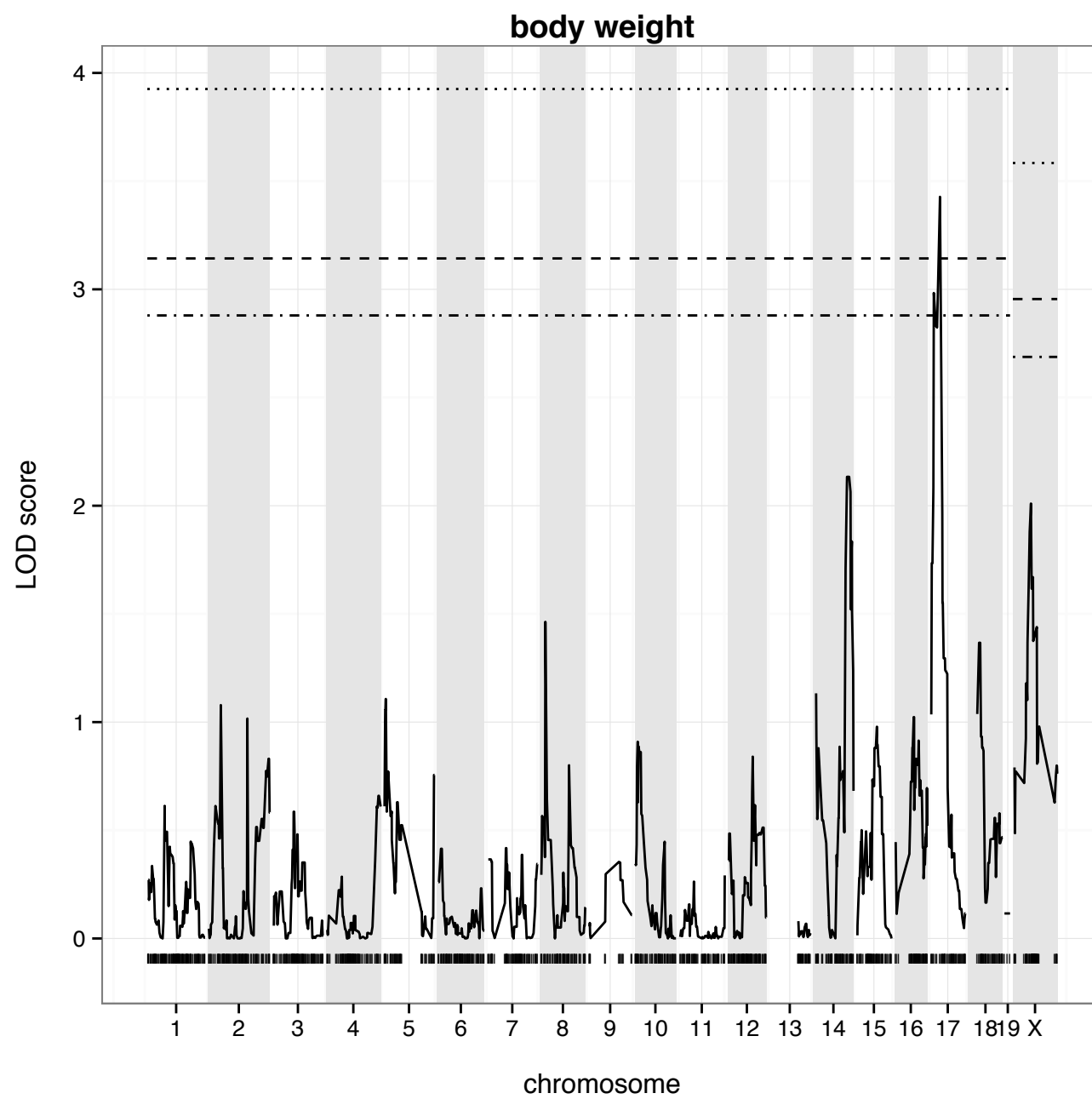

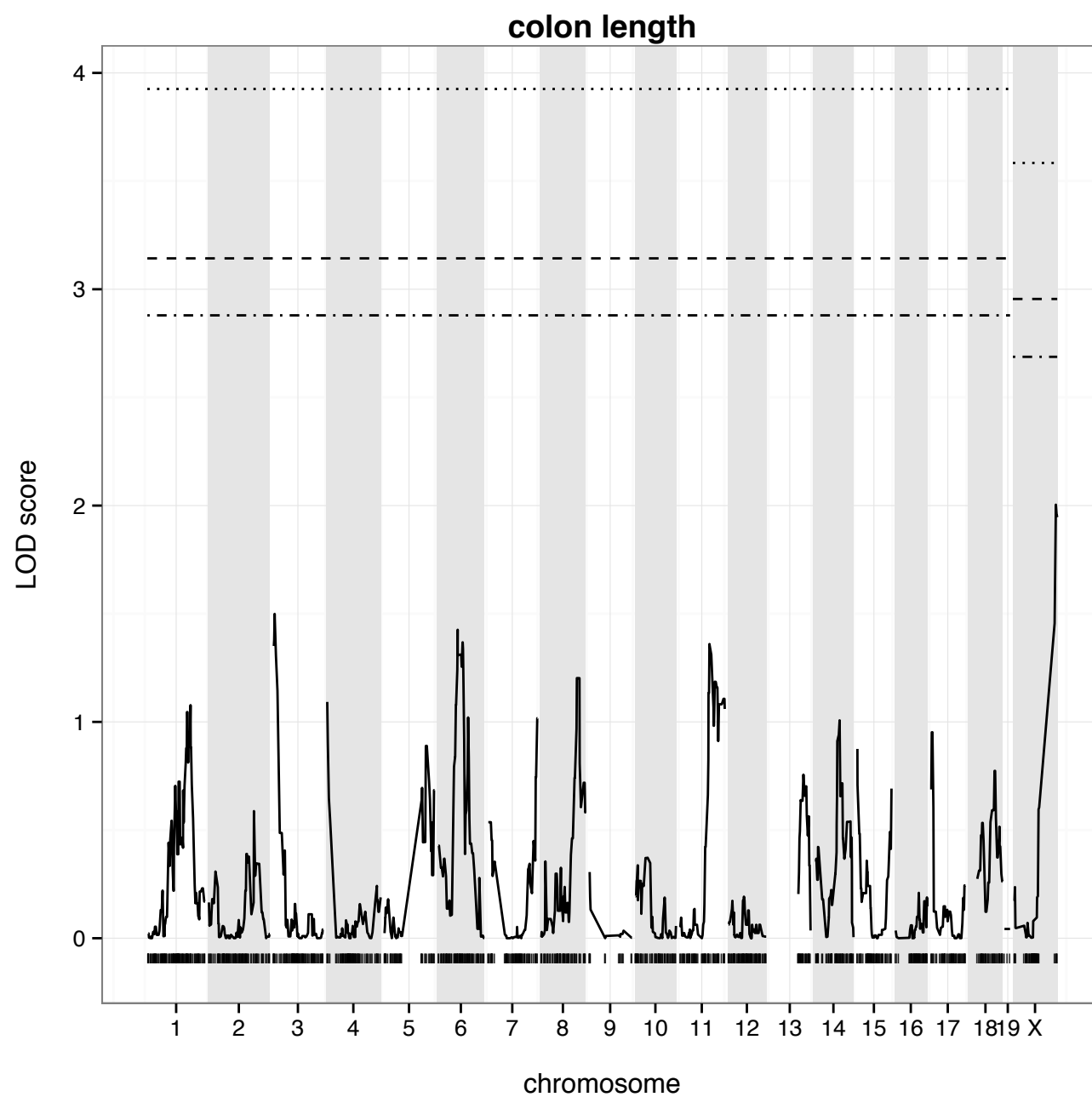

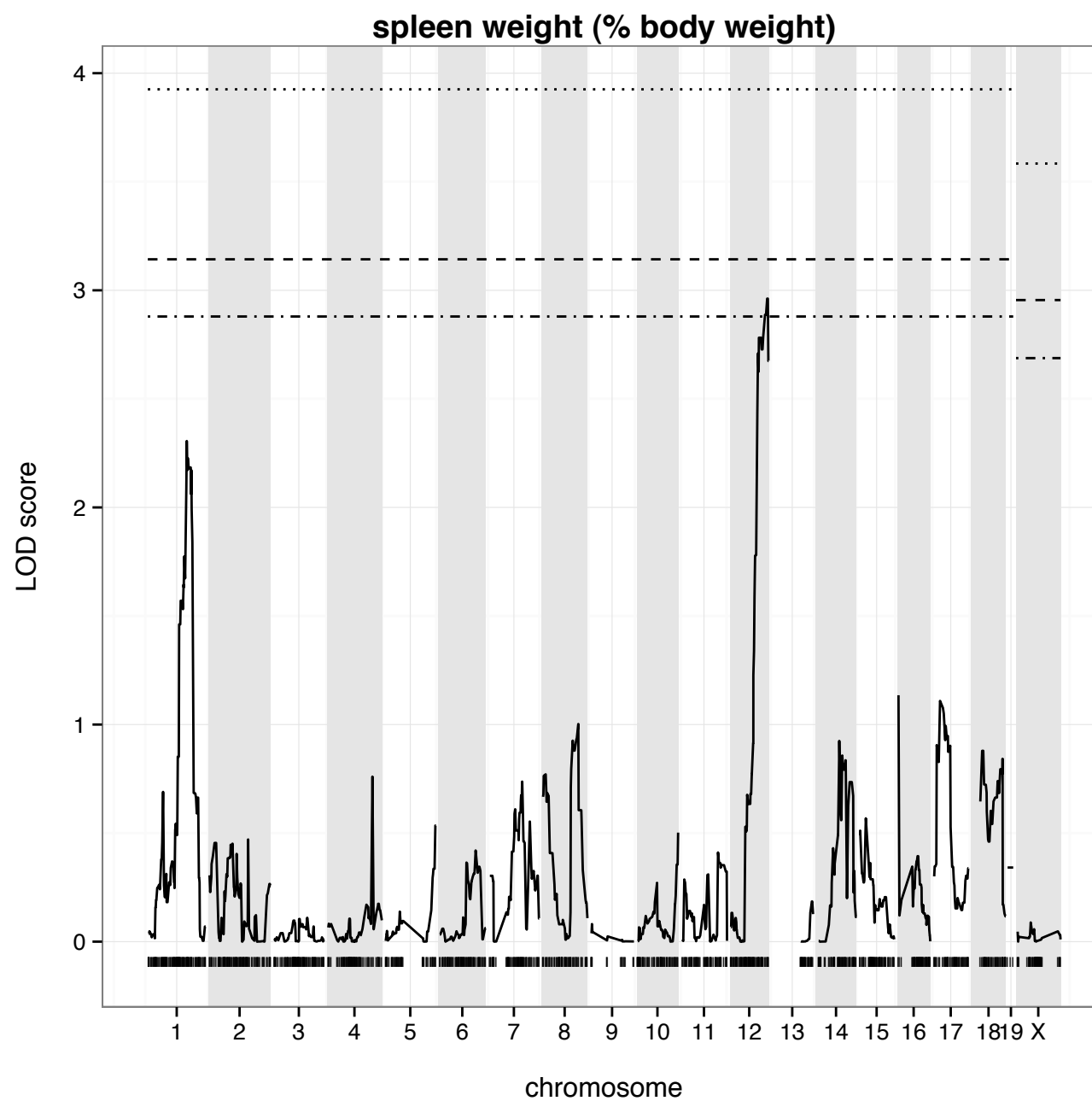

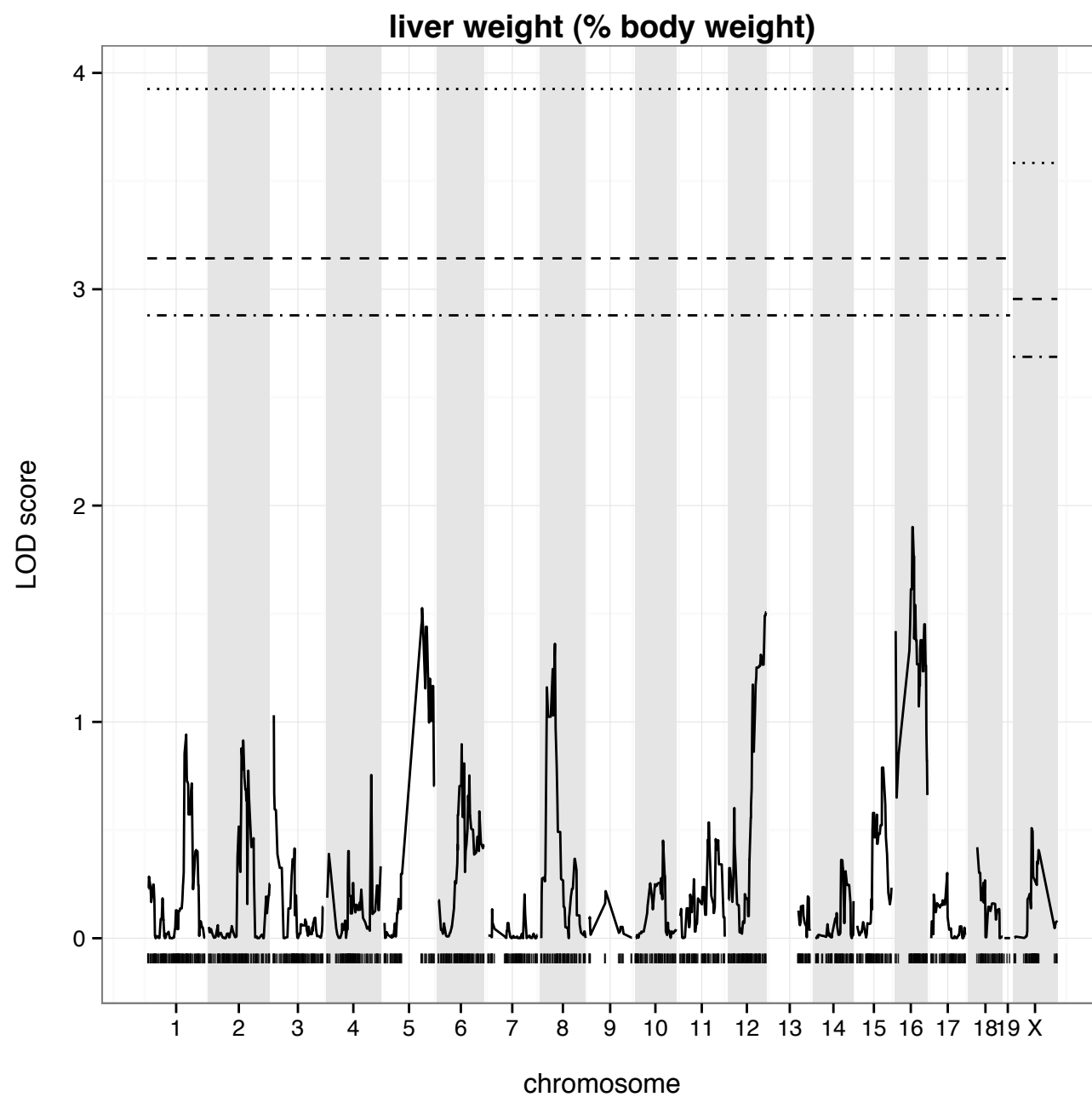

Supplement: Supplementary file 2 — QTL scans for all phenotypes under single-locus model. Significance thresholds (dashed-dotted line, α = 0.10; dashed line, α = 0.05; α = 0.01) were derived from 1,000 permutations, performed separately for the autosomes and for the X chromosome. Vertical hashes along abscissa represent marker positions. (PDF 262 kb) [file 335_2013_9499_MOESM2_ESM.pdf]

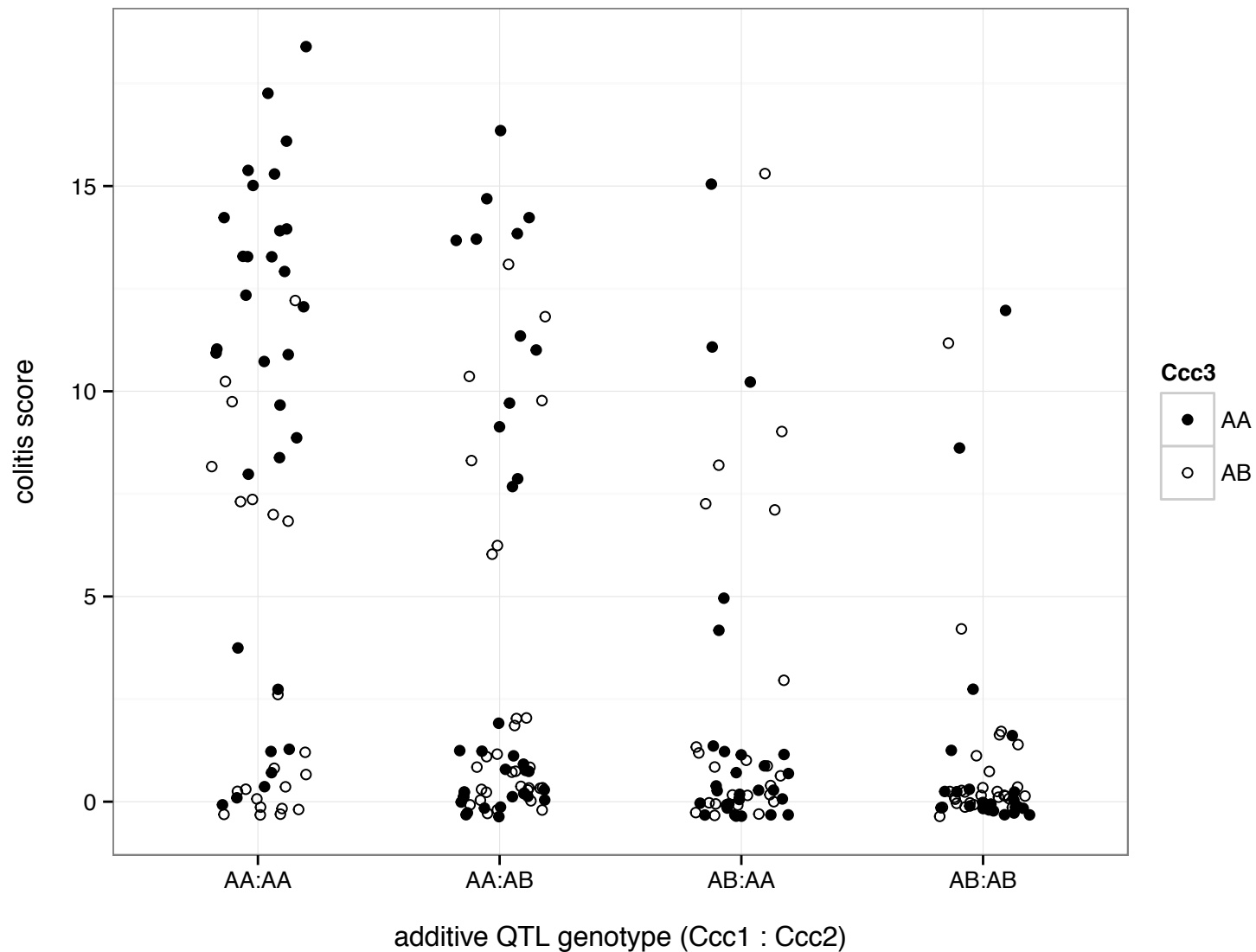

Supplement: Supplementary file 3 — Residual phenotypic variation within genotype classes at Ccc1, Ccc2 and Ccc3. Colitis score (y-axis) within two-locus genotype defined by Ccc1 and Ccc2, coded as AA (homozygous; CC011/CC011) and AB (heterozygous; B6/CC011). Filled circles denote individuals homozygous at the epistatic locus Ccc3 and open circles denote individuals heterozygous at Ccc3. (PDF 33 kb) [file 335_2013_9499_MOESM3_ESM.pdf]
